# Application of permanents of square matrices for DNA identification in multiple-fatality cases

**DOI:** 10.1186/1471-2156-14-72

**Published:** 2013-08-21

**Authors:** Maiko Narahara, Keiji Tamaki, Ryo Yamada

**Affiliations:** 1 Unit of Statistical Genetics, Center for Genomic Medicine, Graduate School of Medicine, Kyoto University, 53 Shogoin Kawahara-cho, Sakyo-Ku, Kyoto, Japan; 2 Department of Forensic Medicine and Molecular Pathology, Graduate School of Medicine, Kyoto University, Yoshida-Konoe-Cho, Sakyo-Ku, Kyoto, Japan

**Keywords:** DNA polymorphism, DNA-based identification, Multiple-fatality cases, Permanent of square matrix, Assignment problem

## Abstract

**Background:**

DNA profiling is essential for individual identification. In forensic medicine, the likelihood ratio (LR) is commonly used to identify individuals. The LR is calculated by comparing two hypotheses for the sample DNA: that the sample DNA is identical or related to a reference DNA, and that it is randomly sampled from a population. For multiple-fatality cases, however, identification should be considered as an assignment problem, and a particular sample and reference pair should therefore be compared with other possibilities conditional on the entire dataset.

**Results:**

We developed a new method to compute the probability via permanents of square matrices of nonnegative entries. As the exact permanent is known as a #P-complete problem, we applied the Huber–Law algorithm to approximate the permanents. We performed a computer simulation to evaluate the performance of our method via receiver operating characteristic curve analysis compared with LR under the assumption of a closed incident. Differences between the two methods were well demonstrated when references provided neither obligate alleles nor impossible alleles. The new method exhibited higher sensitivity (0.188 vs. 0.055) at a threshold value of 0.999, at which specificity was 1, and it exhibited higher area under a receiver operating characteristic curve (0.990 vs. 0.959, *P* = 9.6E-15).

**Conclusions:**

Our method therefore offers a solution for a computationally intensive assignment problem and may be a viable alternative to LR-based identification for closed-incident multiple-fatality cases.

## Background

DNA profiling is crucial in the identification of human remains, particularly when other physical clues are absent. Currently, the genetic status of an individual is commonly profiled by short tandem repeat (STR) loci. There are two types of approaches to DNA-based identification of an unidentified body: 1) direct matching, and 2) kinship testing. Direct matching is performed when the reference DNA of a victim can be obtained from his/her belongings (direct reference). When direct reference is not available, indirect reference is obtained from the victim’s relatives, and a probability distribution of genotypes is inferred for the victim. In either approach, identification is determined based on the likelihood ratio (LR) between two hypotheses: that the DNA of an unidentified body is that of a particular missing person (MP) (hypothesis *H*_1_), and that it is randomly sampled from a population (hypothesis *H*_2_). This approach with use of STRs has been spread since mid-1990th as a routine use for individual identification. The first application to mass disaster identification was reported in 1997 [1], and DNA identification with STRs became the major tool in mass disaster identification. Although the methodology has been well studied and established for one-to-one identification problem, assignment between many bodies and many families (many-to-many identification) has not been studied well. Potential problems of applying the one-to-one comparison to many-to-many situation are 1) that the confidence in assigning a body to a family is not independent of the confidence in assigning others, 2) that the confidence in assignment is not independent of the number of missing data (such as unrecovered bodies and unreported MPs).

To illustrate the problems, if we assume that the number of victims is two and that the DNAs of two bodies and reference DNAs for two MPs are all available, we can assume with a high degree of confidence that either body 1 or body 2 is MP 1 or MP 2. The conventional LR-based method (LR method) independently compares body 1 to MP 1, body 1 to MP 2, body 2 to MP 1, and body 2 to MP 2. However, these four comparisons are not independent; if the probability of body 1 corresponding to MP 1 is very high, the probability of body 1 corresponding to MP 2 should be very small because only two possibilities are considered for the identity of body 1 and the two probabilities must add up to 1. Another concern with the LR method is as follows. If we consider a LR of 1000 to be sufficiently high to approve the identity of a body, what if the LRs for both of our MPs exceed 1000? In this case, we might decide that the MP with the higher LR is body 1. But what if the LR for MP 1 is 1001, and that for MP 2 is 1002? Should we elect MP 2? When the number of victims is small, for example less than 10, the “best” assignment might be successfully determined by “intelligence”, but this method would fail if the number of victims is as large as 1000 or above. The fact that LR is always independent of the probabilities of other pairs in the data raises another problem in LR: Unavailable data cannot be taken into account. Here, “unavailable data” means unrecovered bodies and MPs with no available reference. As unavailable data are a source of uncertainty, the amount of unavailable data should affect the test result. Note that LR is reduced by considering a prior probability, one divided by the total number of victims, but test results do not differ regardless of whether data are complete or most are absent.

To address these problems, many-to-many approaches need to be developed. Lin et al. [2] proposed a global approach that compares the likelihood of assigning all samples obtained from human remains to all reporting families to the likelihood of the null hypothesis where all the samples were assumed to be obtained randomly from a population, which was computationally easy but not realistic. We propose a new method, called the permanent method, in which we compare two hypotheses: *H*_1_, that the identity of body *i* is MP *j*; and *H*^’^_2_, instead of *H*_2_, that the identity of body *i* is any MP other than MP *j*. Clearly, this comparison is more realistic in that all possible hypotheses involving victims of the same incident are considered, and importantly, these two hypotheses together comprise all possibilities, unlike the LR method; that is, the probabilities for the two hypotheses must add up to 1. Therefore in the example above, if the probability that body 1 is MP 1 is 0.99, the probability that body 1 is MP 2 is 0.01.

We considered identification in multiple-fatality cases as an assignment problem and developed a method to compute the probability that body *i* corresponds to MP *j* conditional on the entire dataset. This approach can be summarized as follows. Assume that every recovered body has been genotyped and that a probability distribution of its genotypes is given. Assume that every reported MP has DNA available for genotyping from a “reference” person (either the victim or a close relative), which in turn can be genotyped to obtain a probability distribution of the genotypes. Using this information, we can compute the matrix *P*, which quantifies the compatibility of the body’s genotype with that of a reference person. The matrix *P* is then used to compute matrix *Q*, which computes the probability that body *i* be assigned to MP *j*. This computation takes into account the number of victims, the number of “unreported” victims, and the number of “unrecovered” bodies. If the resulting probability exceeds a certain threshold *v*, then the body is matched to the person. By employing the threshold-based decision made for each pair of one body and one MP, this approach does not necessarily optimize an assignment as a whole, but uses information that is global.

To handle this problem, data must contain the same number of bodies and MPs both theoretically and technically: Theoretically, the number of bodies (whether or not they were recovered) must be the same as the number of MPs (whether or not they were reported); technically, the algorithm to solve this problem requires a square matrix as described below. Therefore, when some of the bodies are not recovered, then they are assumed to exist, but with a genotype probability distribution that is the same as that of the population. Similarly, when some of the victims are not reported as missing or have no available reference DNA, then they are assumed to exist, but with a genotype probability distribution that is the same as that of the population. The key idea in computing the probability is to find the sum of weights of perfect matching in a bipartite graph, known as the permanent of a square matrix. Exact computation of a permanent is #P-complete [3]. A #P class is a set of counting problems that belongs to nondeterministic polynomial time, and a problem is #P-complete if and only if it is in #P. Because it has been proved that the permanent is #P-complete, exact computation of the permanent is not possible in polynomial time, and algorithms for polynomial time approximation have therefore been proposed [4-7]. We employed the Huber–Law algorithm [6], which is, to the best of our knowledge, currently the fastest algorithm when the matrix is dense. The Huber–Law algorithm is an exact sampling algorithm based on the sequential acceptance/rejection method. The accuracy of estimation depends on the acceptance number obtained from an iteration process, and thus when the probability of acceptance is small, computation time may increase dramatically. What is particularly relevant in the case of DNA identification is that a permanent becomes extremely small as the total number of victims increases. Computation time for an extremely small permanent estimation has not been investigated in previous studies.

In this paper, we first describe the logic of our method. We then present the results of a computer simulation performed to evaluate the performance of our method compared with that of the conventional LR-based method, along with results showing the influence of the presence of unavailable data, using receiver operating characteristic (ROC) curve analysis, and the results of assessment of the accuracy and processing time of the approximation algorithm in the case of DNA identification.

## Methods

### Notation

Assume that a fatality incident has a total of *n*_*w*_ victims. Assume that their bodies (some of which may not have been recovered) are denoted by *B* = (*b*_*i*_), *i* = 1, 2, ⋯, *n*_*w*_, and the corresponding MPs (some of whom may not have been reported) are denoted by *M* = (*m*_*j*_), *j* = 1, 2, ⋯, *n*_*w*_. Because all bodies may not have been recovered, let *n*_*b*_ ≤ *n*_*w*_ be the number of recovered bodies. Similarly, because some victims of the fatality may not have been reported, let *n*_*m*_ ≤ *n*_*w*_ be the number of reported MPs. A victim is considered “reported” if either a direct or an indirect reference is available for genotyping. There are four possible situations: 1) *n*_*w*_ = *n*_*b*_ = *n*_*m*_, 2) *n*_*w*_ > *n*_*b*_ and *n*_*w*_ = *n*_*m*_, 3) *n*_*w*_ = *n*_*b*_ and *n*_*w*_ > *n*_*m*_, and 4) *n*_*w*_ > *n*_*b*_ and *n*_*w*_ > *n*_*m*_. In cases 2), 3), and 4), unrecovered bodies and/or unreported MPs exist. *β* = (*β*_*i*_), *i* = 1, ⋯, *n*_*w*_ denotes the probability distributions of the genotypes of *n*_*w*_ bodies with the following properties of *β*_*i*_; 1) the number of elements equals to the number of possible genotypes, 2) each element represents the probability of the genotype being consistent with the genotype of *b*_*i*_, 3) the sum of all the elements equals 1, and 4) when the genotype can be determined, one element is 1 and the other elements are 0. The elements of *β*_*i*_ take either one of the two possible forms:

$$\beta_{i}=\left\{\begin{array}{c} \gamma_{b,i},\\ \gamma_{*}, \end{array}\right)\begin{array}{c} i=1,\cdots ,n_{b}\\ i=n_{b}+1,\cdots ,n_{w} \end{array},$$

where *γ*_*b,i*_ denotes the probability distribution of the genotype of *b*_*i*_, given that the DNA from the body is available for genotyping, and γ_*_ is the probability distribution of genotypes in a population, whereby genotypes of *n*_*w*_ – *n*_*b*_ unrecovered bodies are assumed to follow γ_*_.

Similarly, *μ* = (*μ*_*j*_), *j* = 1, ⋯, *n*_*w*_ denotes the probability distributions of the genotypes of *n*_*w*_ MPs, and *μ*_*j*_ has the same properties as *β*_*i*_. The elements of *μ*_*j*_ are also given as follows:

$$\mu_{j}=\left\{\begin{array}{c} \gamma_{m,j},\;j=1,\cdots ,n_{m}\;\\ \gamma_{*},\;j=n_{m}+1,\cdots ,n_{w} \end{array}.\right)$$

Here, *γ*_*m,j*_ is the probability distribution of the genotype of *m*_*j*_, given its reference DNA, and the probability distribution for *n*_*w*_ – *n*_*m*_ unreported MPs is assumed to follow γ_*_; *β*_*i*_ and *μ*_*j*_ can be expressed as a vector in which the value of each element is the probability of each genotype.

We define an *n*_*w*_ × *n*_*w*_ matrix *P* = (*p*_*i,j*_), where *p*_*i,j*_ is the probability that *b*_*i*_ and *m*_*j*_ have an identical genotype, and is calculated as the inner product of *β*_*i*_ and *μ*_*j*_. We also define an *n*_*w*_ × *n*_*w*_ matrix *Q* = (*q*_*i,j*_), where *q*_*i,j*_ is the probability that *b*_*i*_ and *m*_*j*_ are identical conditional on a matrix *P*.

To calculate a matrix *Q* from a matrix *P*, we consider the *permanents of square matrices*[8]. We use perm(*A*) to denote the permanent of an *n* × *n* matrix *A* = (*α*_*i,j*_). The set of permutations on {1, 2, ⋯, *n*} is *S*_*n*_ and is needed to define perm(*A*). Let $S_{n}^{\left(i,j\right)}$ denote a subset of *S*_*n*_, $S_{n}^{\left(i,j\right)}=\left\{\sigma |\sigma \left(i\right)=j\right\}\in S_{n}$, where σ(*i*) denotes the *i*th element of σ. We use *A*_-*i*,-*j*_ to denote the (*n* – 1) × (*n* – 1) matrix obtained by removing the *i*th row and *j*th column from *A*.

For the criterion for assignment based on *Q*, we use *v* ∈ [0, 1]. The output of the permanent method is either “assigned” or “suspended” for each pair of one body and one MP. A *b*_*i*_ and *m*_*j*_ pair are “assigned” when *q*_*i,j*_ exceeds *v*, and therefore we can approve the identity of *b*_*i*_ to be *m*_*j*_. Otherwise, the identity of *b*_*i*_ is “suspended”, i.e., not approved to be *m*_*j*_. *P*, *Q*, and perm(*A*) are defined in the following sections.

### Matrix *P*

Probability *p*_*i,j*_ is calculated as the inner product of *β*_*i*_ and *μ*_*j*_. A matrix *P* consists of four submatrices

Here, p_*,*j*_, *p*_*i*,*_, and p_*,*_ indicate the inner products of γ_*_ and *γ*_*m,j*_, *γ*_*b,i*_ and γ_*_, and two γ_*_, respectively. Moreover, *∂*_1_ is an *n*_*b*_ × *n*_*m*_ matrix; *∂*_2_ is an (*n*_*w*_ – *n*_*b*_) × *n*_*m*_ matrix consisting of identical rows, $\left(p_{*,1}\cdots p_{*,n_{m}}\right)$; *∂*_3_ is an *n*_*b*_ × (*n*_*w*_ – *n*_*m*_) matrix consisting of identical columns, ${\left(p_{1,*}\cdots p_{n_{b},*}\right)}^{T}$; and *∂*_4_ is an (*n*_*w*_ – *n*_*b*_) × (*n*_*w*_ – *n*_*m*_) matrix consisting of identical values, p_*,*_. Each submatrix corresponds to the collection of each of the following four cases:

Case 1. *b*_*i*_ is recovered and *m*_*j*_ is reported.

Case 2. *b*_*i*_ is not recovered and *m*_*j*_ is reported.

Case 3. *b*_*i*_ is recovered and *m*_*j*_ is not reported.

Case 4. *b*_*i*_ is not recovered and *m*_*j*_ is not reported.

Matrix *P* is required to have at least one non-zero values in each row and column to obtain matrix *Q*. However, it can happen that all elements in a row or column are zero because mutations and genotyping errors may cause Mendelian errors between truly related individuals. The problem can be overcome by considering the probabilities of these factors; for example, when the genotyping error rate is 0.05, in *β*_*i*_, the probability of an observed genotype is 0.95, and the total probability of other genotypes is 0.05. By this, any Mendelian errors do not cause zero in matrix *P*. In our simulation, we did not assume mutations and genotyping errors to simplify the comparison with the LR method, but all elements in the matrix *P* were non-zero values because we limited the family types (see Methods “Generating families with status patterns”). Not assuming mutations and genotyping errors favors neither our method nor the LR method because mutation influence on only values of matrix *P*, which is also used in the LR method, but not on computation process from matrix *P* to matrix *Q*.

### Permanent

In this section, we define the permanent and describe the equations that are important for explaining our method.

The permanent of a matrix *A* is defined as follows:

(1) $$\mathrm{perm}\left(A\right)=\sum_{\sigma \in S_{n}}\prod_{k=1}^{n}a_{k,\sigma \left(k\right)}.$$

Each term expresses the likelihood, or weight, of a permutation. The permanent of *A*_–*i*,–*j*_ can be expressed by $S_{n}^{\left(i,j\right)}$ and *α*_*i,j*_ as follows:

(2) $$\mathrm{perm}\left(A_{‒i,‒j}\right)=\frac{\sum_{\sigma \in }{}_{S_{n}^{\left(i,j\right)}}\prod_{k=1}^{n}a_{k,\sigma \left(k\right)}}{a_{i,j}}$$

The numerator is the sum of weights of permutations in which *α*_*i,j*_ is included. Here, perm(*A*_–*i*,–*j*_) is given by canceling *α*_*i,j*_ in all terms, and perm(*A*) can be rewritten by using *A*_–*i*,–*j*_ and *α*_*i,j*_ as follows:

$$\forall i\in \left\{1,2,\cdots ,n\right\}\;\mathrm{perm}\left(A\right)=\sum_{j=1}^{n}a_{i,j}\mathrm{perm}\left(A_{-i,-j}\right),$$

(3) $$\forall j\in \left\{1,2,\cdots ,n\right\}\;\mathrm{perm}\left(A\right)=\sum_{i=1}^{n}a_{i,j}\mathrm{perm}\left(A_{-i,-j}\right).$$

### Matrix *Q*

Assigning each of *B* to exactly one of *M* without selecting the same MP more than once is considered to be an assignment problem. If a certain permutation $\sigma \in S_{n_{w}}$ is adopted, *b*_*i*_ and *m*_*σ* (*i*)_ are paired with each other. That is, if a permutation $\sigma \in S_{n_{w}}^{\left(i,j\right)}$ is adopted, *b*_*i*_ and *m*_*j*_ are always paired with each other. Here, we refine the definition of *q*_*i,j*_ as the ratio of the sum of the likelihoods of $\sigma \in S_{n_{w}}^{\left(i,j\right)}$ to the sum of the likelihoods of $\sigma \in S_{n_{w}}$. By considering Eq. (1) and Eq. (2), we express *q*_*i,j*_ as follows:

(4) $$q_{i,j}=\frac{\sum_{\sigma \in S_{n_{w}}^{\left(i,j\right)}}\prod_{k=1}^{n_{w}}p_{k,\sigma \left(k\right)}}{\sum_{\sigma \in S_{n_{w}}}\prod_{k=1}^{n_{w}}p_{k,\sigma \left(k\right)}}=\frac{p_{i,j}\times \mathrm{perm}\left(P_{-i,-j}\right)}{\mathrm{perm}\left(P\right)}.$$

A matrix *Q* is given by calculating *q*_*i,j*_ for all pairs of *i* and *j*.

Because both the numerator and the denominator of Eq. (4) include the permanent, we apply the following strategy to minimize estimation error. Let *Q*' = (*q*' _*i*,*j*_) denote an *n*_*w*_ × *n*_*w*_ matrix consisting of the numerator of Eq. (4). We avoid using perm(*P*), instead substituting $\sum_{i=1}^{n_{w}}q{'}_{i,j}$ and $\sum_{j=1}^{n_{w}}q{'}_{i,j}$ for perm(*P*) by Eq. (3):

(5) $$q_{i,j}=\min\left[\frac{{q^{\prime }}_{i,j}}{\sum_{i=1}^{n_{w}}{q^{\prime }}_{i,j}},\frac{{q^{\prime }}_{i,j}}{\sum_{j=1}^{n_{w}}{q^{\prime }}_{i,j}}\right]$$

Here, $\sum_{i=1}^{n_{w}}{q^{\prime }}_{i,j}=\sum_{j=1}^{n_{w}}{q^{\prime }}_{i,j}$ up to an approximation error. We take a smaller value for stringency.

### Determining assignment

We determine assignment based on a matrix *Q* and on *v*, a threshold value for approving identification. Thus, we can approve *b*_*i*_ and *m*_*j*_ to be identical if *q*_*i,j*_ > *v*. To avoid confusion, we say that *b*_*i*_ and *m*_*j*_ are *paired* if they are coupled with each other in a permutation *σ*, and that *b*_*i*_ and *m*_*j*_ are *assigned* if *q*_*i,j*_ > *v*. If *q*_*i,j*_ ≤ *v*, we say that the decision for this pair is *suspended*. We assign or suspend all pairs using the identical value of *v*. Typically, an assignment problem is an optimization problem, in which the solution attempts to find a permutation that optimizes an objective function. For the multi-fatality victim identification problem considered here, such a global objective function is not necessarily appropriate. Therefore, we can decide to either assign or suspend each pair based on *v*. This strategy may lead to the result that none of the elements in a row or a column exceed the value of *v*. In this case, we make no assignment for the corresponding body or MP.

When the value of *v* is set above 0.5, a unique solution can be derived for the problem: For a given *b*_*i*_, when *q*_*i,j*_ > *v* > 0.5, for any $j^{\prime }\neq j,q_{i,j^{\prime }}<1-\nu <0.5$ because $\sum_{j=1}^{n_{w}}q_{i,j}=1$. This can be shown by Eq. (3) as follows. Equation (3) indicates that all column totals and row totals of a matrix *Q*′, which is an *n*_*w*_ × *n*_*w*_ matrix consisting of the numerator of Eq. (4), are equal to perm(*P*). Therefore, all column totals and row totals of a matrix *Q*, which is a matrix *Q*′ divided by perm(*P*), are equal to 1. A similar solution can be found for a given *m*_*j*_.

In other words, a matrix *Q* is approximately a doubly stochastic matrix, which is defined as a square matrix of nonnegative real numbers, of rows and columns each summed to 1. Therefore, when one element in a column is larger than 0.5, no other elements in the same column can be larger than 0.5. The same can be said in terms of rows. Therefore, *v* > 0.5 is the condition sufficient to ensure the uniqueness of the solution. Hereafter, we call this method the permanent method.

What value should be used for cutoff depends on situations and criteria conventionally accepted in a society. Typically, in the LR method, LR of 10^6^ is used, which is equivalent to probability of 0.999999. However, the LR method does not tell you the true probability of making a mistake because there are only two possibilities considered in the LR method; 1) the body is obtained from the MP and 2) the body is randomly drawn from the population. On the other hand, the probability given by the permanent method is truly the probability of making a wrong assignment. Therefore, we consider that the permanent method does not need to follow the conventional cutoff. After close discussion with a forensic expert, expecting one mistake in 1000 of the times is stringent enough to discuss the utility of the permanent method.

**Figure 1:**
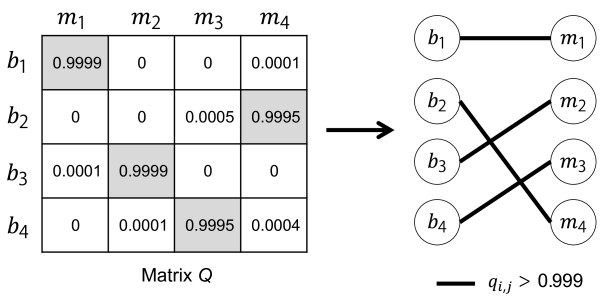
**An example of output of the permanent method.** The matrix on the left represents a matrix *Q*. The bipartite graph on the right corresponds to assignment indicated by the matrix *Q*. The grey cells in matrix *Q* indicate assigned pairs at *v* = 0.999, and the solid lines connect the assigned pairs. For example, the probability of the identity of *b*_1_ being *m*_1_, or *q*_1,1_, is 0.9999, and therefore, *b*_1_ is assigned to *m*_1_. The identity of *b*_1_ cannot be *m*_2_ or *m*_3_, and it can be *m*_4_ but not probable. Note that values in a column and row in the matrix *Q* add up to 1; the matrix *Q* is a doubly stochastic matrix. Because all four bodies are successfully assigned to missing persons, this example obtained a perfect match.

The permanent method is illustrated by Figure 1.

We summarize the permanent method here so that the advantage of the permanent method would be clear. Assuming that we are interested in the probability that the identity of body 1 (*b*_1_) is MP 1 (*m*_1_). *p*_1,1_ is the probability that *m*_1_ has the genotype of *b*_1_ given the reference DNA for *m*_1_, and relationship between *m*_1_ and the reference person. Therefore, *p*_1,1_ is independent of genotypes of other bodies and reference DNAs of other MPs. A likelihood of a possible hypothesis of pairing *n*_*w*_ bodies and *n*_*w*_ MPs is calculated by multiplying elements in matrix *P* that correspond to the paired bodies and MPs. *q*_1,1_ is given by the proportion of the sum of the likelihoods of the hypotheses in which *b*_1_ and *m*_1_ are paired to the sum of the likelihoods of all possible hypotheses. By using matrix *Q* instead of matrix *P*, a probability of *b*_1_ corresponding to *m*_1_ influences the probabilities of *b*_1_ corresponding other MPs. For example, in matrix *P*, two elements in the same row may have high probability such as *p*_1,1_ = 0.95, and *p*_1,2_ = 0.98; *p*_1,2_ is only slightly higher than *p*_1,1_, so we would not determine the identity of *b*_1_. Matrix *Q* is computed by considering all the other elements in matrix *P*. And it may result in *q*_1,1_ = 0.0001 and *q*_1,2_ = 0.9999 depending on other elements in matrix *P*. What makes this happen? It can happen when, *p*_2,1_ is much higher than *p*_1,1_, for example *p*_2,1_ = 0.9999999999, and all the other elements in the first column is 0 or very small. In this case, matrix *Q* reflects this logic: *b*_2_ is assigned to *m*_1_, and therefore, *b*_1_ must not be *m*_1_, and therefore, *b*_1_ is assigned to *m*_2_.

### Likelihood ratio and posterior probability

The LR of *b*_*i*_ corresponding to *m*_*j*_ is defined as *p*_*i,j*_ divided by the genotype frequency of *b*_*i*_ in a population,

(6) $$\mathrm{LR}=\frac{\beta_{i}\cdot \mu_{j}}{\beta_{i}\cdot \gamma_{*}}.$$

The numerator of Eq. (6) corresponds to the likelihood that hypothesis *H*_1_ is true (the DNA of an unidentified body is that of a particular MP), and the denominator corresponds to the likelihood that *H*_2_ is true (the DNA is randomly sampled from a population). Therefore, the numerator is equal to *p*_*i,j*_ defined in the permanent method. The difference between the two methods becomes apparent after we calculate matrix *P*: Unlike in the permanent method, in the LR method, the matrix *P* is divided by the genotype frequency of the corresponding body. For example, *p*_1,j_, *j* = 1,2,…, *n*_*w*_ are divided by the genotype frequency of *b*_1_.

In multiple-fatality cases, the prior odds are commonly considered as 1/(*n*_w_ – 1), and according to the Bayesian theorem, the posterior odds are given by the product of LR and the prior odds, *r* = LR/(*n*_w_ – 1). We use the posterior odds, *r*, for test values of the LR method.

### Assessment of performance

Probability and odds are interchangeable, and therefore we can use the same assignment criterion, *v*, to compare *q*_*i,j*_ and *r*. We can assess the performance of discrimination between *identical* and *non-identical* pairs by using the area under a ROC curve (AUC) [9]. We state that a body and MP pair is identical when the body is that of the MP, and that the pair is non-identical when the body is that of another MP. A ROC curve plots sensitivity against 1 − specificity, and the AUC is commonly used to evaluate the performance of a measure. Larger values of AUC indicate higher performance.

In our case, sensitivity is defined as the ratio of the number of assigned truly identical pairs to the total number of truly identical pairs, and specificity is defined as the ratio of the number of suspended truly non-identical pairs to the total number of truly non-identical pairs. AUC is calculated by summing the areas of trapezoids formed between two adjacent cut-off points (trapezoidal method), and its confidence interval (CI) is estimated by the DeLong method [10]. Two ROC curves are compared by testing their AUCs with a nonparametric method described by DeLong et al. [10].

In the LR method, two or more MPs can be assigned to a body by any cutoff *v*. In a practical situation, the identity of the body needs to be determined to one, otherwise, it remains undetermined. However, here we consider that, the LR method assigns all pairs that exceeded the cutoff because decision for this case in practice is made with combinatorial information other than DNA, and because the ROC analysis is to assess discriminating performance of an index itself. The same strategy was applied to the permanent method when the cutoff value was set to be *v* ≤ 0.5.

### Simulating a population

We performed a computer-based simulation to assess the performance of the permanent method for DNA profiles of 15 STR loci available in the ABI AmpFISTR Identifiler® PCR Amplification Kit (Applied Biosystem, Foster City, CA, USA). Theoretically, as the number of markers increases, the performance of both the permanent and the LR methods increases. However, both methods assume independence of markers. That is a possible limitation to the number of independent markers, and currently identification by a large number of loci has not been applied to practice. The amelogenin locus was excluded from the study because it is used for sex determination. Although two loci are located within chromosome 2 and another two loci within chromosome 5, we assumed that all 15 loci are independent because the influence of the recombination rate is not an issue when assessing the performance of measures. We used previously reported allele frequencies in a Japanese population [11].

**Figure 2:**
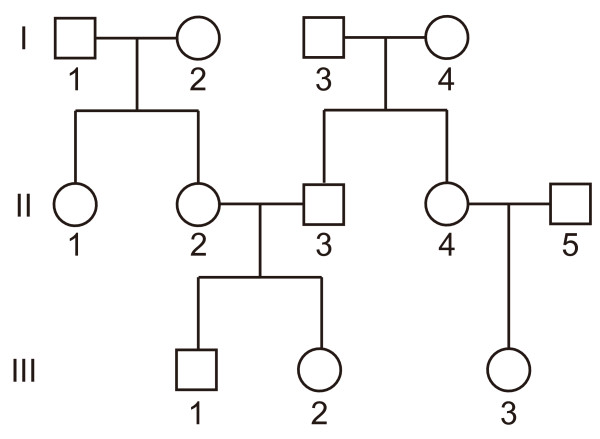
**An example of simulated family pedigrees.** All simulated families have this form of tree. Gender pattern in generation II and III may differ.

DNA profiles of 12,500 families were simulated for the 15 STR loci. Simulated pedigree trees are shown in Figure 2. Genotypes of founders (I-1, I-2, I-3, I-4, and II-5) were randomly provided under the assumption of the Hardy–Weinberg equilibrium, and the genotypes of other family members were determined stochastically based on founders’ genotypes without mutations. Each individual has two types of information: One is his/her genotype, and the other concerns the relationship with family members. We describe the simulated 12,500 families as *pooled* families.

### Generating families with status patterns

For each individual, we set one of three statuses, *missing*, *typed*, or *not-typed*, without allowing for multiple MPs in a family. *Missing* status means that a body is being sought for the person, *typed* status is assigned when the DNA of the person is available for genotyping as a reference for the MP, and *not-typed* means that the family members’ DNA is not available. The status pattern of a family indicates which person is assigned to which status. For example, if in a family of father, mother, and a child, the child is missing, and the mother provides her DNA as a reference, while the DNA of the father is unavailable, then the status pattern of this family is that the child is *missing*, the mother is *typed*, and the father is *not-typed*. The simulated genotype of the MP serves as the genotype of an unidentified body. We assume that no error occurs in genotyping, and thus, one element of *β*_*i*_ is 1 with all the others 0. A vector *μ*_*j*_, which is to be estimated from the reference DNA, was computed by an algorithm and program implemented by the authors [12].

We calculated a matrix *P* between all typed individuals in each family and each body, not between each typed individual and each body. Throughout, we assumed that only kinship testing is employed, because direct matching has almost absolute discriminatory power such as 1 in 2.5 × 10^17^[13]. LR may not be sufficiently high to determine the identity when kinship testing is performed using a reference DNA that provides no obligate alleles or impossible alleles. Obligate alleles are those that an individual must carry and are revealed by the genotypes of his/her parents. Impossible alleles are those that cannot be carried without mutations; they are revealed, for example, when a child has an allele that is carried by none of his/her parents. In other words, therefore, these references that were considered in our simulation do not reveal Mendelian error in any genotypes. We used 10 status patterns that provide neither obligatory alleles nor impossible alleles for MPs. Otherwise, both the LR and the permanent method were highly accurate with little difference between them. Columns headed “Family types” in Table 1 list these 10 types of families. The position of each individual in the pedigree tree is expressed as a combination of generation and a number shown below each symbol in Figure 2 (e.g., I-2 indicates a person numbered 2 in generation I).

**Table 1:** Area under curve observed via computer simulation

**Family types**			**Permanent**			**LR**			
	**M**	**T**	**AUC (95% CI)**^**a**^	**Mean (SD)**^**b**^	**Se/sp**^**c**^	**AUC (95% CI)**^**a**^	**Mean (SD)**^**b**^	**Se/sp**^**c**^	***P***
1	III-1	III-2	1.000 (1.000-1.000)	1.000 (0.000)	0.990/1	0.999 (0.998-0.999)	0.999 (0.001)	0.445/1	1.6E-05
2	I-1	II-3,III-1	0.999 (0.998-0.999)	0.988 (0.005)	0.285/1	0.976 (0.969-0.984)	0.976 (0.021)	0.045/1	1.2E-10
3	I-1	III-1	0.982 (0.977-0.987)	0.982 (0.020)	0.038/1	0.944 (0.932-0.956)	0.945 (0.030)	0.005/1	1.9E-13
4	III-1	I-1	0.982 (0.977-0.987)	0.982 (0.020)	0.035/1	0.944 (0.932-0.956)	0.945 (0.030)	0.005/1	2.1E-13
5	II-1	III-1	0.975 (0.969-0.980)	0.974 (0.020)	0.030/1	0.933 (0.920-0.947)	0.933 (0.029)	0.010/1	1.2E-16
6	III-1	II-1	0.974 (0.969-0.980)	0.974 (0.021)	0.032/1	0.933 (0.920-0.947)	0.933 (0.029)	0.010/1	1.3E-16
7	III-1	I-1,I-3	1.000 (1.000-1.000)	0.999 (0.002)	0.673/1	0.988 (0.984-0.992)	0.988 (0.011)	0.118/1	2.1E-08
8	I-1	I-3,I-4,III-1	0.995 (0.993-0.997)	0.994 (0.009)	0.168/1	0.962 (0.953-0.972)	0.962 (0.020)	0.033/1	2.8E-15
9	I-1	I-3,III-1	0.990 (0.987-0.993)	0.989 (0.014)	0.078/1	0.951 (0.940-0.961)	0.951 (0.026)	0.015/1	9.6E-20
10	III-1	III-3	0.821 (0.799-0.843)	0.818 (0.074)	0.000/1	0.788 (0.764-0.812)	0.788 (0.068)	0.000/1	2.5E-08
Mix			0.990 (0.987-0.993)	0.988 (0.013)	0.188/1	0.959 (0.950-0.968)	0.958 (0.022)	0.055/1	9.6E-15

*M* missing, *T* typed, Permanent: permanent method, *LR* LR method.

^a^Area under curve (AUC) of pooled results of 20 datasets for each family type (estimated 95% confidence interval (CI)).

^b^Mean of AUC (standard deviation (SD)) of 20 datasets for each family type.

^c^Sensitivity and specificity at threshold 0.999.

### Data for ROC analyses

For comparison with the LR method, we assumed a complete dataset, i.e., *n*_*w*_ = *n*_*b*_ = *n*_*m*_. Complete data are required to compare two methods under the same condition because the LR method does not take unavailable data into account. We generated 20 datasets, each of which consisted of 20 families randomly drawn from the pooled families. Uniform data were generated by assigning the same status pattern to all 400 families, 20 datasets × 20 families. Because we used 10 types of pedigrees, there were 200 datasets, 10 types × 20 sets, in total. We used the same 400 families for all family types, so that performance could be compared using a set of families derived from the same set of founders. For comparison in a more realistic situation, 20 mixed datasets were generated by assigning status patterns randomly chosen from the 10 types with an equal probability to the same 400 families. The same sets of families were used again for the same reason. To summarize the results of 20 datasets, we pooled 8,000 (20 datasets × 20 bodies × 20 families) values of matrix *Q* obtained from the 20 datasets, and a ROC curve was drawn using all the values. We describe the AUC obtained from this ROC curve as pooled AUC. Results for uniform datasets are summarized in Table 1 for each family type and shown with mean AUC (standard deviation (SD)), pooled AUC (95% CI), and *P* values of the DeLong test for the alternative hypothesis that the pooled AUC of the permanent method is greater than that of the LR method. Similarly for the mixed data, the results were summarized with mean AUC (SD), pooled AUC of all 20 datasets, and *P* values (Table 1).

For assessing the influence of unavailable data, 20 families were randomly drawn from the pooled families. The bodies of MPs of these families were assumed to have been recovered, and we call this data *complete part*. Appropriate numbers of bodies and/or families were randomly drawn and added to the data. These additional bodies or families did not have identical counterparts, and we call this data *additional part*. Data for the additional part were drawn 10 times to obtain 10 datasets, each with the same complete part. We simulated three types of situations: 1) *n*_*b*_ < *n*_*m*_ = *n*_*w*_,  2) *n*_*m*_ < *n*_*b*_ = *n*_*w*_,  and 3) *n*_*b*_, *n*_*m*_ < *n*_*w*_. For situation 1, family data were added to the complete part, and body data that were unavailable were completed using γ_*_. For situation 2, body data were added, and family data that were unavailable were completed via γ_*_. For situation 3, equal numbers of families and bodies were added, and the remainder were completed with γ_*_. A matrix *Q* was calculated using the entire dataset, and only values of the complete part were used to draw the ROC curve. This is because the aim of this simulation was to compare performance using complete data to demonstrate how the amount of unavailable data influences the performance. The reason for this is as follows. The number of identical pairs depends on the size of the complete part, i.e., 20 in our simulation. On the other hand, the number of non-identical pairs increases according to the size of the incomplete part, because all entries in the incomplete part are incorrect matches. Therefore, the number of non-identical pairs differs between a complete dataset and an incomplete dataset. This means that the influence of one incorrect assignment on specificity differs between the complete dataset and the incomplete dataset; the influence is weaker in the incomplete dataset because the number of non-identical pairs is larger in the incomplete dataset than that in the complete dataset. Therefore, the performance would be naturally different when all entries in both complete and incomplete parts are taken into account. However, we are interested in how the performance is changed by the presence of the incomplete part, compared to the performance in the complete dataset. Therefore, we performed a ROC analysis for the complete part in the incomplete dataset. On the other hand, the results of sensitivity and specificity at the threshold *v* = 0.999 were obtained from all the entries in both the complete and incomplete parts.

### Approximation of the permanent of the square matrix

We employed an algorithm to approximate a permanent of a nonnegative square matrix, as described by Huber and Law [6]. Briefly, two parameters, *δ* and *ϵ*, define the accuracy of the approximation as follows: For any *ϵ* ≥ 0 and *δ* ∈ (0, 1], the permanent of a square matrix with arbitrary nonnegative entries can be approximated within a factor of 1+ *δ* with probability at least 1–*ϵ*. That is, *δ* gives the lower and upper limits of the estimate, and *ϵ* gives the probability that the estimate falls within the limits. The algorithm is based on a sequential acceptance/rejection procedure, in which the ratio of the number of accepted trials (the acceptance number) to the total number of trials is used to approximate a permanent. The two accuracy parameters define the acceptance number as *k* = 14*δ*^–2^ln(2/*ϵ*). Because both small *δ* and *ϵ* increase *k*, a large acceptance number means that accuracy is high. The sequential acceptance/rejection iteration process ends when a preset *k* is achieved. We used *δ =* 0.5 and *ϵ* = 0.0001, and thus *k* = 555, when not specified. We chose these values to maximize accuracy within a practical computational cost range. We verify these values next under “Assessment of approximation accuracy and computation time” and in the Results under “Accuracy parameters of approximation and computation time”.

### Assessment of approximation accuracy and computation time

To assess accuracy and computation time in our case, test matrices were generated using values obtained from a matrix *P* of the mixed datasets used in the ROC analyses. To obtain practical matrices, each matrix contained exactly one value for an identical pair in each row and column, and values for non-identical pairs in the remaining elements. We prepared matrices of sizes 20 and 30 for assessing accuracy, and matrices of sizes of 10, 20, 30, and 40 for assessing computation time. We calculated the permanents of these matrices 100 times each, with combinations of two accuracy parameters, *δ* = (0.5, 1) and *ϵ* = (0.1, 0.01, 0.001, 0.0001, 0.00001). For *δ* = 0.5 and *ϵ* = 0.0001, we also simulated a worst-effect scenario, in which approximation errors affect the test result in the worst manner. The worst-effect scenario is defined as that in which, for a body, $q_{\mathrm{ij}}^{'}$ for an identical pair is approximated to be equal to the lower limit, and in which $q_{\mathrm{ij}}^{'}$ of the most probable pair among non-identical pairs in the same row for the body in matrix *Q*′ is approximated to be equal to the upper limit. Because *ϵ* = 0.0001 is sufficiently low, we consider that no more than one non-identical pair in each row of matrix *Q*′ is approximated to be equal to the upper limit. Here, the most probable non-identical pair is not the result of an approximated permanent, but is obtained if it were possible to exactly compute the permanent. Therefore, in the worst-effect scenario, the probability of the most probable non-identical pair is overestimated, the probability of the identical pair is underestimated, and thus these errors maximize the influence on specificity and sensitivity. We assessed the performance of the permanent method for a case in which this worst effect of errors occurred to all bodies in a dataset. We assumed that the matrix *Q*′ of the same mixed dataset used in the ROC analysis was obtained from an exact computation of permanents, and we generated matrix *Q′* for the worst case from the assumed exact results, and computed matrix *Q* with the same method as described in the Methods under “Matrix *Q*”.

Mean computation time was obtained by approximating three times for each matrix size and for the same combinations of parameters.

### Computation environment and software

Genotype simulation, computation of conditional probabilities and LRs, evaluation of performance, and assessment of accuracy and processing time were performed with R v2.13.1 [14]. The computation environment was as follows: Vostro 260S (Dell), 64-bit Windows 7 operating system, Intel Core i5-2400 CPU (3.10 GHz). We computed a probability distribution of genotypes of a MP, *μ*_*j*_, a matrix *P* and LR using an algorithm proposed and implemented by the present authors [12] to compute kinship applicable to general forms of pedigrees, including large extended families. ROC analysis was performed with the pROC package [15] in R.

## Results

### Comparison of permanent method and LR-based method

**Figure 3:**
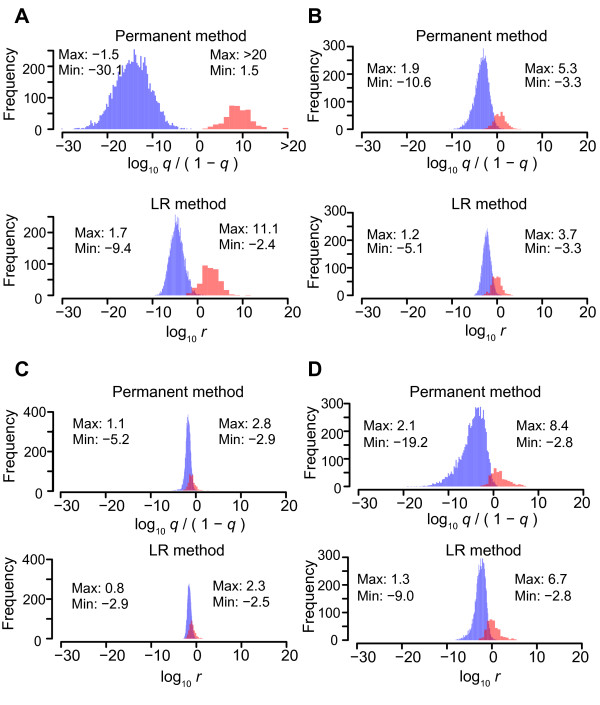
**Distributions of conditional probabilities of permanent method and posterior odds of LR method.** Distributions of identical pairs and non-identical pairs are shown in red and blue, respectively. Minimal and maximal values of the distributions are shown for non-identical pairs (left) and for identical pairs (right). Probabilities obtained with permanent method are shown as odds. Values are obtained from 20 uniform datasets of either type 1 **(A)**, type 4 **(B)**, type 10 **(C)**, or mixed **(D)**.

First, we assessed performance on uniform datasets to compare performance for each family type. Figure 3A–C shows distributions of probabilities obtained with the permanent method and posterior odds obtained with the LR method for uniform datasets of family types 1, 4, and 10 respectively, corresponding to family type in Table 1.

**Figure 4:**
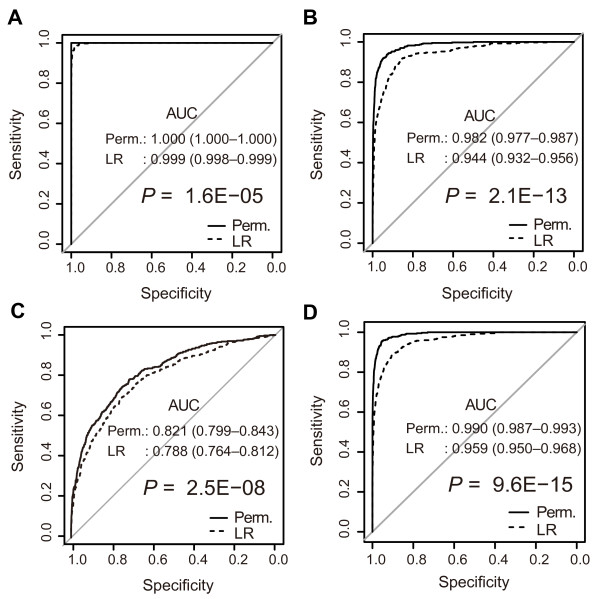
**ROC curves of pooled results obtained from 20 datasets for each family type. (A)** Uniform data of type 1. **(B)** Uniform data of type 4. **(C)** Uniform data of type 10. **(D)** Mixed data. Discriminant performance was compared between the permanent method (solid line) and the LR method (dashed line). AUC (95% confidence interval (CI)) and *P* values of the DeLong test are shown.

The histograms indicate that the distributions of identical pairs and non-identical pairs overlap less when family types 1 and 4 were tested with the permanent method as compared with the LR method. Notably, when tested with the permanent method, the distributions of identical pairs and non-identical pairs show no overlap for family type 1, or sibship tests. Therefore, the two distributions are more clearly separated when the permanent method is used. For family type 10, or between-cousins test, however, the distributions do not differ much between the two methods. Figure 4A–C shows ROC curves of pooled results of 20 uniform datasets for the same three family types. For all the three types, the permanent method significantly outperformed the LR method in terms of AUC (*P* = 1.6E-05, 2.1E-13, and 2.5E-08 for type 1, 4, and 10, respectively). Figure S1 and S2 show histograms and ROC curves for other family types, and Table S1 lists statistics for each dataset (see Additional file 1: Figure S1, Additional file 2: Figure S2, Additional file 3: Table S1). The permanent method exhibited significantly better performance than the LR method for all family types in terms of AUC. When the threshold value was set to 0.999, a realistic threshold in practice, the permanent method exhibited higher sensitivity than the LR method for all family except type 10, while specificity was 1 with both methods for all family types.

**Figure 5:**
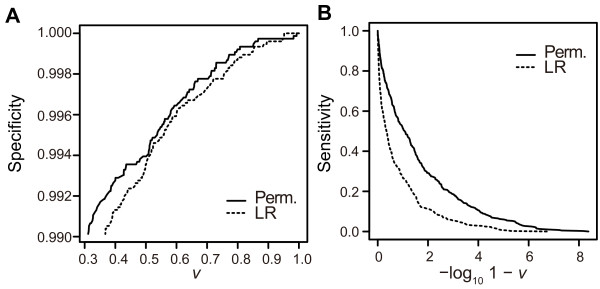
**Specificity and sensitivity resulting from the mixed datasets. (A)** Specificity is plotted against threshold values, *v*. Specificity of 0.99 or above are shown. **(B)** Sensitivity is plotted against **-**log_10_(1-*v*).

Next, we assessed performance on mixed datasets for comparison in a more practical situation. Counts of simulated family types included in each dataset are listed in Table S2 (see Additional file 3: Table S2). Figure 3D shows the distributions of identical pairs and non-identical pairs. The overlapping area is smaller when the permanent method is used. Figure 4D shows ROC curves for pooled results of 20 mixed datasets. Table 1 and Table S3 show statistics for the pooled results of all datasets and the result of each dataset, respectively (see Additional file 3: Table S3). The permanent method exhibited significantly better performance (AUC: 0.990 vs. 0.959, *P* = 9.6E-15). This suggests that the permanent method should be available for use in practical situations. In a practical situation, achieving high specificity is considered to be more important than optimizing both sensitivity and specificity. In this paper, we use *v* = 0.999, which means that, when the probability of a body and missing-person pair exceeds 0.999, the sum of probabilities of the other pairs in the same column or row is 0.001, and thus we consider 0.999 to be a highly reliable threshold (our choice of the threshold value is discussed further in the Discussion). With *v* = 0.999, the permanent method exhibited higher sensitivity than the LR method (0.188 vs. 0.055), while specificity was 1 with both methods (Table 1, Figure 5A, Figure 5B).

**Figure 6:**
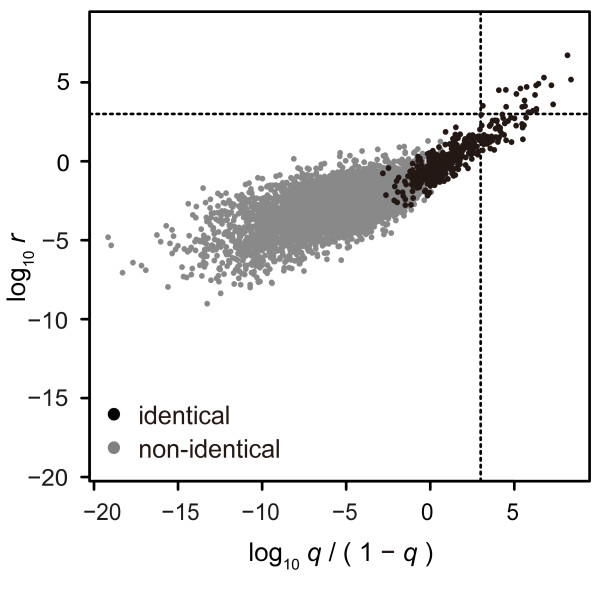
**Posterior odds of LR method plotted against conditional probabilities of permanent method for mixed datasets.** Distribution of test values of the permanent method and the LR method resulting from the mixed pedigree datasets is shown for identical pairs (black), and non-identical pairs (gray). Probabilities obtained with the permanent method are shown as odds. Dashed lines indicate the level equivalent to 0.999. Top-right and bottom-left regions indicate that the judgments of the two methods were consistent, and top-left and bottom-right regions indicate that the judgments between the two methods differed.

ROC analysis indicated some difference between the two methods in terms of overall performance. We also compared differences in the judgment of each pair. Figure 6 shows the distributions of values of the permanent method and LR method. Dashed lines indicate the level of *v* = 0.999, with the four regions divided by the dashed lines demonstrating the following judgments:

•top right: assigned by both methods,

•top left: assigned by LR method and suspended by permanent method,

•bottom left: suspended by both methods, and

•bottom right: assigned by permanent method but suspended by LR method.

Top-right and bottom-left regions indicate that the judgments of the two methods were consistent, while the top-left and bottom-right regions indicate that the judgments between the two methods differed. Table 2 shows pair counts classified into each region at threshold values 0.999 and 0.9999. At the level of 0.999, neither method assigned any non-identical pairs. The permanent method assigned 75 truly identical pairs, among which the LR method failed to assign 53 pairs. The LR method assigned 22 truly identical pairs, all of which were assigned by the permanent method. At the level of 0.9999, the permanent method assigned 43 truly identical pairs, among which the LR method failed to assign 31 pairs. The LR method assigned 12 pairs, all of which were assigned by the permanent method. Figure 5A and Figure 5B plot specificity and sensitivity, respectively, at various values of *v*. Specificity curves are similar with both methods, and the permanent method and LR method reached the specificity of 1 at 0.993 and 0.952, respectively. On the other hand, sensitivity differed between the two methods, with the permanent method exhibiting a higher sensitivity at any value of *v*. These results suggest that, at the level of 0.999 or higher, both methods safely discriminate non-identical pairs, and the permanent method demonstrates higher sensitivity. As shown in Figure 3D, Figure 6 also indicates that the test values of non-identical pairs are distributed more widely when the permanent method is used.

**Table 2:** Counts of assignment status

**Judgment**		***v*** **= 0.999**		***v*** **= 0.9999**	
**Permanent**	**LR**	**Non-identical**	**Identical**	**Non-identical**	**Identical**
Suspend	Suspend	7600	325	7600	357
	Assign	0	0	0	0
Assign	Suspend	0	53	0	31
	Assign	0	22	0	12

Permanent: permanent method, *LR* LR method, *v*: threshold value.

### Demonstration of how the permanent method performs for incomplete data

Here we demonstrate the performance of the permanent method on incomplete data compared with complete data. Table S4 lists the counts of family types drawn by random sampling in each dataset (see Additional file 3: Table S4). Table 3 lists the mean decrease in AUCs resulting from the complete part of incomplete datasets from AUC of the complete dataset in each situation and each number of additional bodies and/or families. Statistics of each dataset are listed in Table S5 (see Additional file 3: Table S5). Sensitivity and specificity at the level of 0.999 were calculated from all the entries in both complete and incomplete parts, and are listed in Table 3. Note that all the complete parts of the incomplete datasets and the complete dataset include the identical set of bodies and reference families, but conditional probabilities resulting from the permanent method differ because of variation in the additional part. Although the number of total victims is limited in our simulation because of computation time constraints, discussed in the next section, as the amount of additional-part data increased, mean decreases from complete data increased, and sensitivity at *v* = 0.999 showed a tendency to decrease. Even with 10 additional bodies and/or reference families, which means that about 30% of the data are missing, the permanent method still exhibited equal or higher sensitivity at 0.999 and higher AUC compared with the LR results for the corresponding complete data.

**Table 3:** Area under curve of datasets with missing bodies or families

**Data**	**Situation**	**Method**	**Additional data**	**AUC (SD)**^**b**^	**Decrease from complete data (SD)**^**c**^	**Se/sp**^**b**^
Complete^d^		Perm.	0	0.9945		0.20/1
		LR	0	0.9651		0.05/1
Incomplete^a^	1	Perm.	4	0.9937 (0.0005)	0.0008 (0.0005)	0.06/1
		Perm.	10	0.9913 (0.0015)	0.0032 (0.0015)	0.05/1
	2	Perm.	4	0.9928 (0.0018)	0.0017 (0.0018)	0.14/1
		Perm.	10	0.9908 (0.0017)	0.0036 (0.0017)	0.09/1
	3	Perm.	4	0.9920 (0.0016)	0.0025 (0.0016)	0.05/1
		Perm.	10	0.9882 (0.0048)	0.0063 (0.0048)	0.05/1

*SD* standard deviation, *Perm.* permanent method, *LR* LR method, *AUC* area under curve.

^a^Data shown for incomplete cases were results of permanent method.

^b^For incomplete datasets, the complete parts with the same 20 victims were used to calculate AUC. Sensitivity and specificity were calculated from all entries in both the complete part and the incomplete part. Mean values of 10 datasets for each situation and each number of additional data are shown for AUC.

^c^Mean decrease from AUC of complete data measured with the permanent method.

^d^Data shown for complete cases were obtained using only a complete part with the permanent method (Perm.) and LR method.

### Accuracy parameters of approximation and computation time

**Figure 7:**
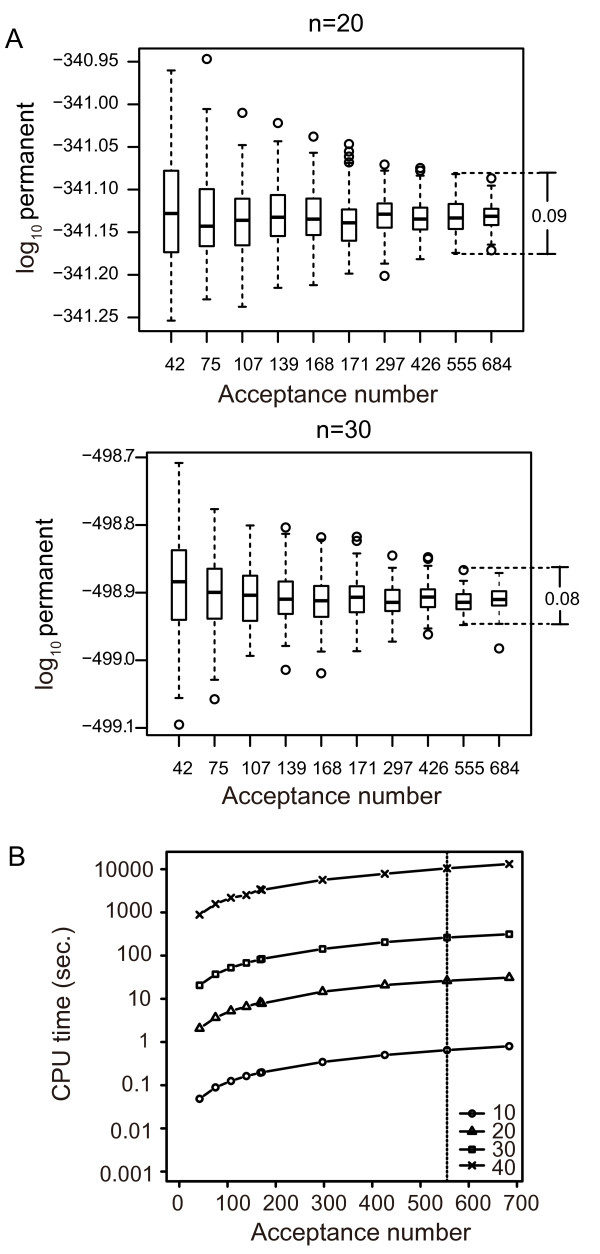
**Accuracy and computation time of approximation of permanent. (A)** Distribution of 100 estimates of a permanent for each matrix size 20 (top) and 30 (bottom). Acceptance number *k* = 555 is equivalent to the accuracy used in the simulation to assess performance. Bars on the right indicate the difference between the highest and lowest estimates at *k* = 555. **(B)** Computation time and acceptance number. CPU time (seconds) is plotted against the acceptance number for matrices of size 10 (circle), 20 (triangle), 30 (square), and 40 (cross). Dashed lines indicate the acceptance number, *k* = 555, used in the simulation to assess performance.

We investigated the distribution of the estimates of the permanent in the setting of DNA identification. Figure 7A shows the distribution of estimates obtained from 100 estimates of permanents plotted against the acceptance number, *k*. Table S6 shows values of *δ* and *ϵ* and their corresponding values of *k* (see Additional file 3: Table S6). Under the condition used to assess performance with the above results (*k* = 555), the differences between the minimal and the maximal estimates were 0.09 and 0.08 in a log scale for the matrix sizes 20 and 30, respectively, which means that the maximal estimate fell on the minimal estimate multiplied by 10^0.09^ = 1.23 and 10^0.08^ = 1.20, respectively (Figure 7A). When simulating the worst case, we observed a decrease in sensitivity from 0.19, which was the sensitivity obtained from the mixed datasets shown in Table 1, to 0.18 (four pairs assigned in the assumed exact result failed to exceed *v* = 0.999 in the worst case), and no changes in specificity (1 even in the worst case). Figure S3 shows that the assumed exact test results and the test results in the worst case only differ slightly; test values for identical pairs in the worst case are slightly lower than the corresponding assumed true values, and test values for non-identical pairs in the worst case are slightly higher than the corresponding assumed true values (see Additional file 4: Figure S3). Figure 7B plots computation time against acceptance number. When the matrix size was 40, computation time increased dramatically, and it took approximately 11,000 seconds = 3 hours to obtain the acceptance number used in the simulation.

## Discussion

The individual identification of unidentified bodies is an important issue. Developments in DNA-based techniques have provided strong evidence for personal identification. Despite progress in genotyping, challenging cases are still encountered, such that LR fails to provide strong evidence because kinship between sample and reference DNA itself is not close. If it occurs in a single-fatality case, DNA would not be the first-line evidence. In the case of multiple fatalities, approaches optimized for multiple-fatality cases may overcome this problem to some extent. LR-based identification is optimal when comparison is only possible with a random person from a population, i.e., a single-fatality case. In multiple-fatality cases, however, comparison can be performed with other DNA obtained from the same case. Currently, identification in multiple-fatality cases is based on a conventional LR weighted by prior odds. Although the LR method can take the total number of victims into account via the prior odds, it still compares each pair only with the population where the possible identity of a body can be limited to one of MPs. The new approach we have described in this paper considers the identification problem as an assignment problem and provides the probability for a particular pair of sample and reference DNA conditional on the entire observed data. Because the permanent method considers all assignment hypotheses, it can be reasonably speculated that, given pairs assigned with high confidence, conditional probabilities of non-identical pairs decrease and those of other identical pairs increase. Thus, we expect the permanent method to be capable of discriminating between identical and non-identical pairs that cannot be clearly discriminated with LR.

In a separate study, the distribution of combined sibship index (CSI) with 15 STR loci was investigated, and it was found that the distribution of CSI for siblings overlapped with that for random pairs; it was also found that 1.3–1.6% of siblings had CSI less than 1, and 1.4–1.9% of random pairs had CSI greater than 1 [16]. Thus, a sibship testing, or type 1 in our simulation, in multiple-fatality cases would be more difficult, where prior odds reduce LR.

The results of our computer simulation show that the performance of the permanent method is significantly higher than that of the LR method. In the case of individual identification, high specificity is required even at the cost of sensitivity, because finding an incorrect identity is a crucial problem. Thus, we also focused on sensitivity and specificity at *v* = 0.999 and higher, and the permanent method displayed higher performance in this range. However, the choice of threshold value in practice is rather arbitrary depending on the situation and the criteria conventionally accepted in a society. After close discussion with a forensic expert, we decided to use *v* = 0.999 and *v* = 0.9999 to discuss our results. For other threshold values, Figure 5A and Figure 5B show sensitivity and specificity according to the values of threshold in our simulation. Uniform datasets were tested to assess performance in terms of difficulty levels for a problem. The three family types, 1, 4, and 10, were expected to represent levels of relatively easy, difficult, and very difficult, respectively. Because type 1 corresponds to a sibship testing, the fact that the permanent method resulted in no overlap between distributions of identical pairs and non-identical pairs, and that non-identical pairs showed distributions with larger variance, indicate well the greater discriminating power of the permanent method (Figure 3A).

In this paper, we used only cases in which references provide neither obligate alleles nor impossible alleles; that is, any genotypes are consistent without assuming mutation. This is because the current genotyping system provides a discriminating power strong enough to overcome the theoretical problem in the LR method, and both methods perform highly with almost no difference between them (data not shown). Thus, the power of the permanent method may be maintained up to some extent, but not as far as between cousins (although the ROC analysis indicated that the permanent method performed better in terms of AUC, the sensitivity and specificity at the practical threshold did not differ between the two methods). In practice, it is clear that we should attempt to collect reference DNA from relatives who are as closely related as possible, in order to obtain obligate alleles. If this is not possible, it is important to consider whether references provide impossible alleles. Consider a situation in which the DNA of the parents of a MP is not available but that of all four grandparents is available, but for financial reasons we can only type two persons. Two grandparents on the same side may provide impossible alleles, but two from each side do not. In our simulation, these two situations led to a substantial difference in results; when two grandparents on the same side were used, both the permanent and the LR method were more powerful in determining the identities (data not shown).

Importantly, the permanent method can take uncertainty into account. Two situations lead to uncertainty: the presence of unavailable data, and the number of unknown victims. As simulated in this paper, the former can be considered by the permanent method. Although the latter is not demonstrated, the same strategy can be applied: We would set an expected total number of victims and complete the unavailable data with γ_*_. As we do not know the exact number of victims, the expected number would be arbitrary to some extent. Judgment in the LR method does not change no matter how much unavailable data exist, whereas assignment by the permanent method becomes more difficult with more unavailable data. Therefore, the test value of the permanent method appears to reflect our assignment confidence better than that of the LR method.

Although the theoretical basis of our approach is reasonable and adequate, the approach is computationally challenging, because the permanent method requires estimation of the permanents *n*_*w*_ × *n*_*w*_ times. Computation time can be the primary cause of limitation. The most efficient algorithm for the exact permanent requires Θ(*n*2^*n*^) arithmetic operation. It has been proven that exact computation of the permanent is a #P-complete problem, even for 0,1 matrices, and thus computation in polynomial time is not possible [3]. Since then, researchers have focused on approximation algorithms. Currently, to the best of our knowledge, the approximation approach described by Huber and Law [6] is the fastest algorithm when the problems become dense. Huber and Law achieved *O*(*n*^4^ log *n*) expected running time, though there is no *a priori* bound on the running time for matrices with arbitrary nonnegative entries. In our study, we implemented the Huber–Law methodology to simulate our approach. In our simulation environment, we experienced a dramatic increase in computation time when the matrix size was 40. Therefore, the permanent method is currently practically applicable to the assignment of relatively small numbers of victims in a closed incident. However, we are hopeful about the possibility of extending the application, even to large open incidents, because of advances in two areas: (1) approximation algorithms and combination theory, and (2) computer performance. On the first point, approximation algorithms for the permanent of a matrix are a vigorously studied field of mathematics. Recent improvements include those of Jerrum et al. (2004) [7], Bezáková et al. (2006) [4], and Huber and Law (2008) [6]; the computational complexities of these methods are *O*(*n*^10^ (log*n*)^3^), *O*(*n*^7^ (log*n*)^4^), and *O*(*n*^4^ log *n*), respectively. These dramatic decreases in complexity have been experienced only in the last ten years, and further developments can therefore be expected. Second, we expect that the permanent method will enjoy more extended application in the near future because computer performance is continually improving. For these reasons, we believe that there is merit in our new idea for DNA identification in multiple-fatality cases, even with current computational limitations.

Because computation of the permanent method depends on approximation algorithms of a matrix, we must describe the algorithm-specific consideration. The Huber–Law algorithm defines two accuracy parameters *δ* and *ϵ*. The acceptance number *k*, given by *δ* and *ϵ*, only influences the computation process. This means that various sets of *δ* and *ϵ* that give the same *k* are mutually interchangeable. However, here we suggest using values of *δ* and *ϵ* to better understand the accuracy. Smaller values of *δ* and *ϵ* give a more accurate approximation. We verified that *δ* = 0.5 and *ϵ* = 0.0001 can give sufficiently accurate test results for our simulation condition, and thus we suggest using *δ* ≤ 0.5 and *ϵ* ≤ 0.0001.

To reduce computation time, a Monte Carlo method can be applied where the total number of iterations is set instead of *k*. However, with a Monte Carlo method, we must take care how many accepted trials are obtained after the iteration. In the case of DNA identification, we found lower probabilities in obtaining one accepted trial as the matrix size increased. In this case, the Monte Carlo method may result in small values of *k*, and estimates may become highly unreliable, as shown in Figure 7A. Therefore, the Monte Carlo method is not recommended for practical DNA identification problems.

## Conclusions

The permanent method provides further evidence for identification in terms of conditional probability. We have shown that this method is capable of detecting identical pairs of low LR and is highly robust in terms of specificity. With two methods used in combination, DNA-based identification may exhibit higher performance. It is also important that the permanent method is capable of weighting the presence of unavailable data, unlike the LR method. Currently, the permanent method is computationally limited to relatively small datasets obtained from closed incidents.

## Competing interests

The authors declare that they have no competing interests.

## Authors’ contributions

MN participated in the design of the study, wrote the program, carried out the computer-simulation and the statistical analysis, and drafted the manuscript. KT conceived of the study, and supervised the forensic aspect of the study. RY developed the algorithm, wrote the program, designed the study, and helped to draft the manuscript. All authors read and approved the final manuscript.

## Supplementary Material

Additional file 1 **Distributions of conditional probabilities of permanent method and posterior odds of LR method.** Distributions of identical pairs and non-identical pairs are shown in red and blue, respectively. Probabilities obtained with the permanent method are shown as odds. Values are obtained from 20 uniform datasets of family types 2, 3, 5, 6, 7, 8, or 9. Family types are defined in Table 1. Click here for file

Additional file 2 **ROC curves of pooled results obtained from 20 datasets for each family type.** ROC curves of test results for family types 2, 3, 5, 6, 7, 8, and 9 are shown. Discriminant performance was compared between the permanent method (solid line) and the LR method (dashed line). AUC (95% confidence interval (CI)) and *P* values of the DeLong test are shown. Family types are defined in Table 1. Click here for file

Additional file 3 **Supplementary tables (Table S1 – S6). Table S1.** Results of the ROC analysis for each dataset for the uniform-pedigree analysis. **Table S2.** Counts of family types that were randomly sampled for the mixed-pedigree analysis. **Table S3.** Results of the ROC analysis for each dataset for the mixed-pedigree analysis. **Table S4.** Counts of family types in the complete part and additional parts that were randomly sampled for the analysis of the incomplete datasets. **Table S5.** Results of the ROC analysis for each dataset for the analysis of the incomplete datasets. **Table S6.** Acceptance numbers corresponding to values of *δ* and *ϵ*. Click here for file

Additional file 4 **Conditional probabilities obtained with permanent method for assumed exact results and worst-scenario results.** Conditional probabilities obtained from worst-effect approximation errors are plotted against those obtained from assumed exact computation of permanent for mixed datasets (shown in a log_10_ scale). Click here for file
