# Supplementary material for: Application of permanents of square matrices for DNA identification in multiple-fatality cases
Source: BMC Genet. 2013 Aug 21;14:72. doi: 10.1186/1471-2156-14-72 (PMC3765903; doi:10.1186/1471-2156-14-72)
Supplement: Additional file 1 — Distributions of conditional probabilities of permanent method and posterior odds of LR method. Distributions of identical pairs and non-identical pairs are shown in red and blue, respectively. Probabilities obtained with the permanent method are shown as odds. Values are obtained from 20 uniform datasets of family types 2, 3, 5, 6, 7, 8, or 9. Family types are defined in Table 1. [file 1471-2156-14-72-S1.pdf]

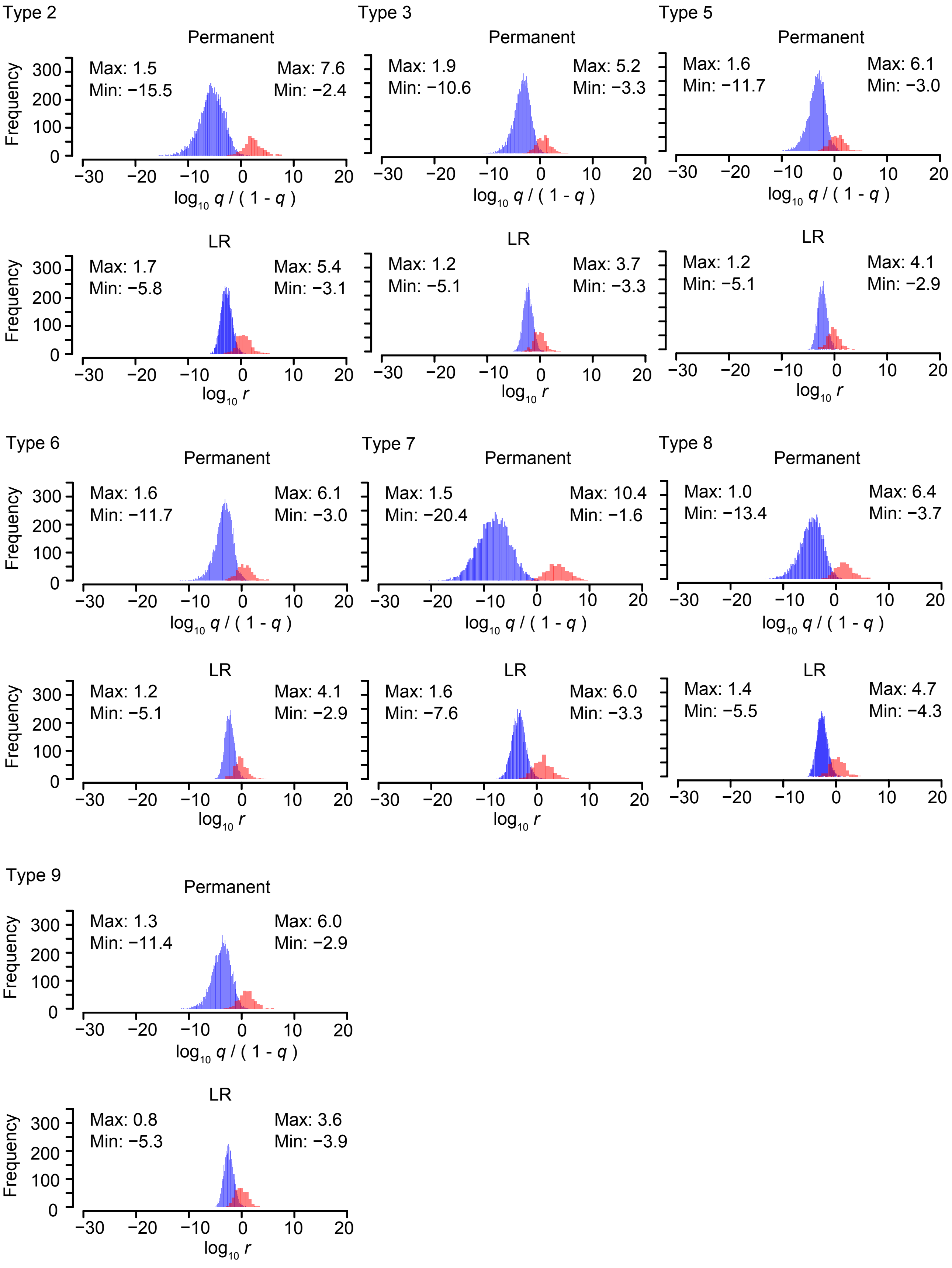

Type 2
Permanent
Frequency
Max: 1.5
Min: −15.5
Max: 7.6
Min: −2.4
log10 q / (1 - q)
LR
Max: 1.7
Min: −5.8
Max: 5.4
Min: −3.1
log10 r
Type 3
Permanent
Max: 1.9
Min: −10.6
Max: 5.2
Min: −3.3
log10 q / (1 - q)
LR
Max: 1.2
Min: −5.1
Max: 3.7
Min: −3.3
log10 r
Type 5
Permanent
Max: 1.6
Min: −11.7
Max: 6.1
Min: −3.0
log10 q / (1 - q)
LR
Max: 1.2
Min: −5.1
Max: 4.1
Min: −2.9
log10 r
Type 6
Permanent
Max: 1.6
Min: −11.7
Max: 6.1
Min: −3.0
log10 q / (1 - q)
LR
Max: 1.2
Min: −5.1
Max: 4.1
Min: −2.9
log10 r
Type 7
Permanent
Max: 1.5
Min: −20.4
Max: 10.4
Min: −1.6
log10 q / (1 - q)
LR
Max: 1.6
Min: −7.6
Max: 6.0
Min: −3.3
log10 r
Type 8
Permanent
Max: 1.0
Min: −13.4
Max: 6.4
Min: −3.7
log10 q / (1 - q)
LR
Max: 1.4
Min: −5.5
Max: 4.7
Min: −4.3
Type 9
Permanent
Max: 1.3
Min: −11.4
Max: 6.0
Min: −2.9
log10 q / (1 - q)
LR
Max: 0.8
Min: −5.3
Max: 3.6
Min: −3.9
log10 r
