# Supplementary figures and images for: Application of permanents of square matrices for DNA identification in multiple-fatality cases
Source: BMC Genet. 2013 Aug 21;14:72. doi: 10.1186/1471-2156-14-72 (PMC3765903; doi:10.1186/1471-2156-14-72)

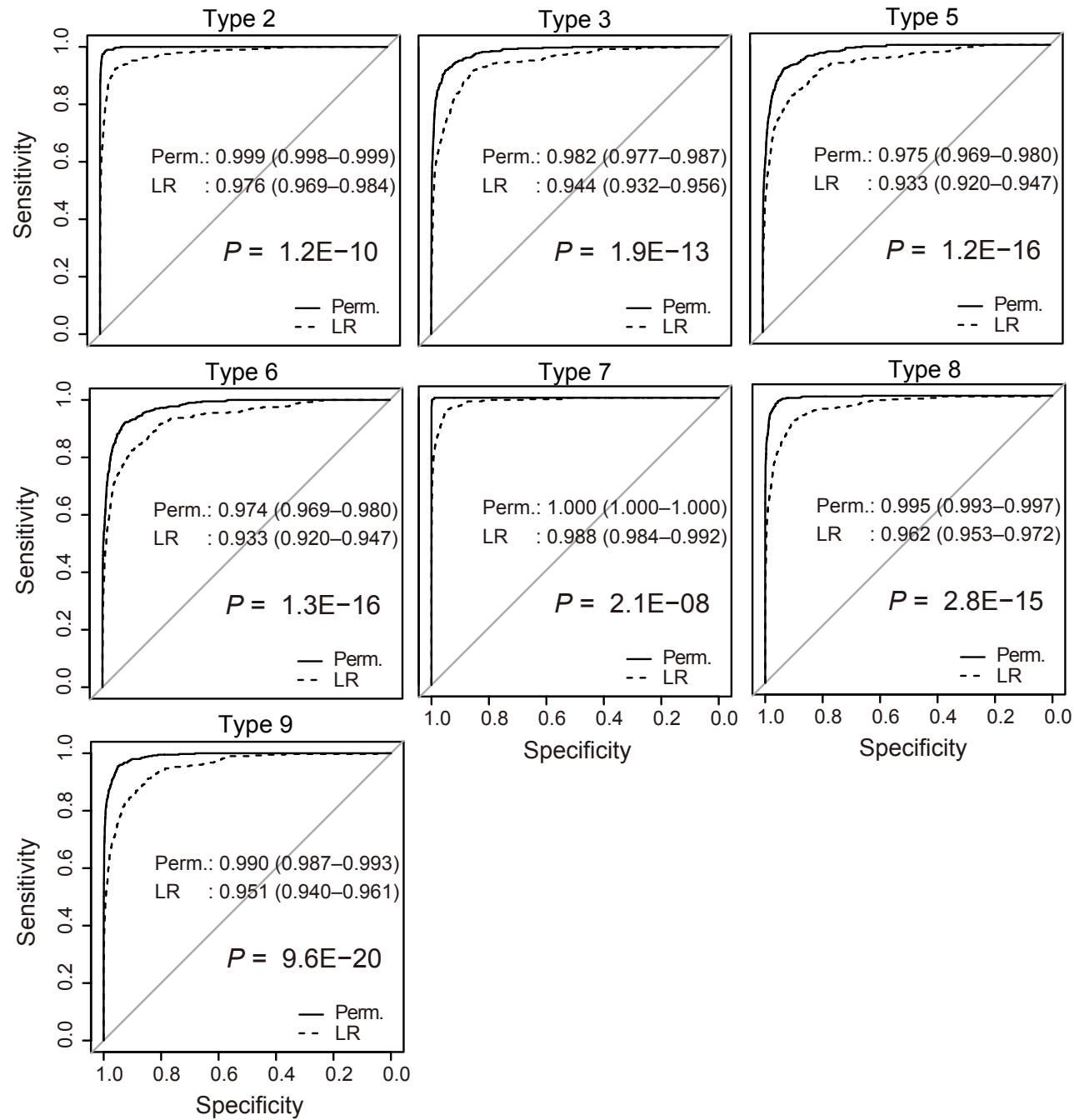

Supplement: Additional file 2 — ROC curves of pooled results obtained from 20 datasets for each family type. ROC curves of test results for family types 2, 3, 5, 6, 7, 8, and 9 are shown. Discriminant performance was compared between the permanent method (solid line) and the LR method (dashed line). AUC (95% confidence interval (CI)) and P values of the DeLong test are shown. Family types are defined in Table 1. [file 1471-2156-14-72-S2.pdf]

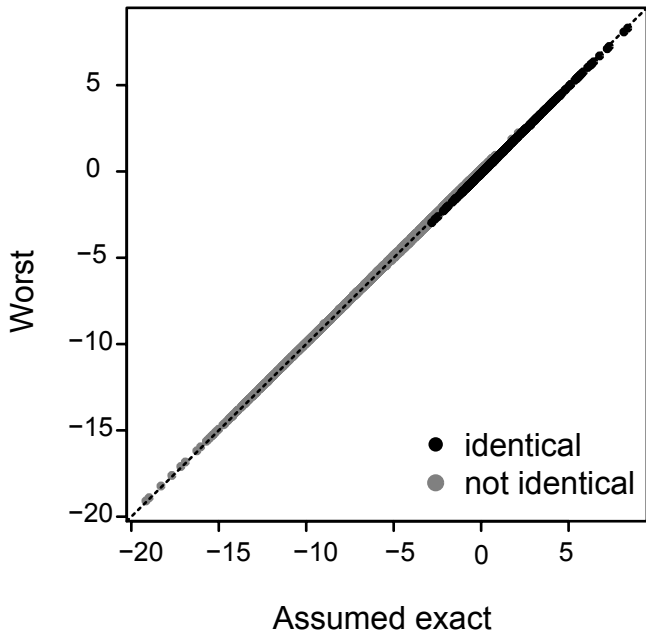

Supplement: Additional file 4 — Conditional probabilities obtained with permanent method for assumed exact results and worst-scenario results. Conditional probabilities obtained from worst-effect approximation errors are plotted against those obtained from assumed exact computation of permanent for mixed datasets (shown in a log10 scale). [file 1471-2156-14-72-S4.pdf]
