# Supplementary material for: Application of permanents of square matrices for DNA identification in multiple-fatality cases
Source: BMC Genet. 2013 Aug 21;14:72. doi: 10.1186/1471-2156-14-72 (PMC3765903; doi:10.1186/1471-2156-14-72)
Supplement: Additional file 3 — Supplementary tables (Table S1 – S6). Table S1. Results of the ROC analysis for each dataset for the uniform-pedigree analysis. Table S2. Counts of family types that were randomly sampled for the mixed-pedigree analysis. Table S3. Results of the ROC analysis for each dataset for the mixed-pedigree analysis. Table S4. Counts of family types in the complete part and additional parts that were randomly sampled for the analysis of the incomplete datasets. Table S5. Results of the ROC analysis for each dataset for the analysis of the incomplete datasets. Table S6. Acceptance numbers corresponding to values of δ and ϵ. [file 1471-2156-14-72-S3.pdf]

Table S1: Result of each uniform dataset

		Permanent				LR				DeLong	
Family [b]	Dataset	CI low	AUC	CI high	Sens./spec.[a]	CI low	AUC	CI high	Sens./spec.[a]	Statistic	P
1	1	1.0000	1.0000	1.0000	0.9 / 1	0.9972	0.9991	1.0000	0.55 / 1	0.94	0.175
	2	1.0000	1.0000	1.0000	1 / 1	0.9965	0.9987	1.0000	0.5 / 1	1.18	0.119
	3	1.0000	1.0000	1.0000	1 / 1	1.0000	1.0000	1.0000	0.2 / 1	0.00	0.500
	4	1.0000	1.0000	1.0000	1 / 1	0.9978	0.9992	1.0000	0.6 / 1	1.11	0.133
	5	1.0000	1.0000	1.0000	1 / 1	0.9938	0.9974	1.0000	0.3 / 1	1.43	0.077
	6	1.0000	1.0000	1.0000	0.9 / 1	0.9867	0.9949	1.0000	0.2 / 1	1.23	0.109
	7	1.0000	1.0000	1.0000	1 / 1	0.9961	0.9984	1.0000	0.55 / 1	1.35	0.088
	8	1.0000	1.0000	1.0000	1 / 1	0.9887	0.9951	1.0000	0.35 / 1	1.47	0.070
	9	1.0000	1.0000	1.0000	1 / 1	0.9991	0.9997	1.0000	0.7 / 1	0.82	0.205
	10	1.0000	1.0000	1.0000	1 / 1	0.9942	0.9976	1.0000	0.5 / 1	1.36	0.087
	11	1.0000	1.0000	1.0000	1 / 1	0.9985	0.9995	1.0000	0.25 / 1	1.01	0.155
	12	1.0000	1.0000	1.0000	1 / 1	0.9951	0.9979	1.0000	0.1 / 1	1.49	0.069
	13	1.0000	1.0000	1.0000	1 / 1	0.9956	0.9984	1.0000	0.45 / 1	1.11	0.134
	14	1.0000	1.0000	1.0000	1 / 1	0.9985	0.9995	1.0000	0.55 / 1	1.01	0.155
	15	1.0000	1.0000	1.0000	1 / 1	0.9975	0.9991	1.0000	0.5 / 1	1.16	0.123
	16	1.0000	1.0000	1.0000	1 / 1	0.9991	0.9997	1.0000	0.6 / 1	0.82	0.207
	17	1.0000	1.0000	1.0000	1 / 1	0.9972	0.9989	1.0000	0.6 / 1	1.18	0.120
	18	1.0000	1.0000	1.0000	1 / 1	1.0000	1.0000	1.0000	0.45 / 1	0.00	0.500
	19	1.0000	1.0000	1.0000	1 / 1	0.9987	0.9996	1.0000	0.45 / 1	0.88	0.190
	20	1.0000	1.0000	1.0000	1 / 1	1.0000	1.0000	1.0000	0.5 / 1	0.00	0.500
2	1	1.0000	1.0000	1.0000	0.25 / 1	0.9898	0.9953	1.0000	0.05 / 1	1.69	0.045
	2	1.0000	1.0000	1.0000	0.2 / 1	0.9638	0.9797	0.9957	0 / 1	2.49	0.006
	3	0.9985	0.9995	1.0000	0.55 / 1	0.9890	0.9945	0.9999	0.1 / 1	1.92	0.027
	4	0.9748	0.9879	1.0000	0.15 / 1	0.8614	0.9295	0.9975	0 / 1	1.85	0.032
	5	0.9697	0.9854	1.0000	0.1 / 1	0.8588	0.9299	1.0000	0 / 1	1.89	0.029
	6	1.0000	1.0000	1.0000	0.5 / 1	0.9889	0.9943	0.9998	0.05 / 1	2.02	0.022
	7	0.9805	0.9903	1.0000	0.2 / 1	0.8864	0.9446	1.0000	0.15 / 1	1.72	0.043
	8	0.9995	0.9999	1.0000	0.15 / 1	0.9526	0.9737	0.9948	0 / 1	2.45	0.007
	9	1.0000	1.0000	1.0000	0.35 / 1	0.9687	0.9862	1.0000	0.05 / 1	1.55	0.061
	10	1.0000	1.0000	1.0000	0.15 / 1	0.9710	0.9842	0.9974	0.1 / 1	2.34	0.010
	11	0.9936	0.9971	1.0000	0.05 / 1	0.9480	0.9739	0.9999	0 / 1	1.85	0.032
	12	0.9985	0.9995	1.0000	0.45 / 1	0.9800	0.9889	0.9979	0.1 / 1	2.37	0.009
	13	1.0000	1.0000	1.0000	0.15 / 1	0.9350	0.9693	1.0000	0 / 1	1.75	0.040
	14	1.0000	1.0000	1.0000	0.45 / 1	0.9924	0.9966	1.0000	0.05 / 1	1.62	0.052
	15	0.9995	0.9999	1.0000	0.2 / 1	0.9635	0.9825	1.0000	0 / 1	1.80	0.036
	16	0.9982	0.9993	1.0000	0.45 / 1	0.9404	0.9750	1.0000	0.1 / 1	1.41	0.079
	17	1.0000	1.0000	1.0000	0.35 / 1	0.9796	0.9900	1.0000	0.1 / 1	1.88	0.030
	18	1.0000	1.0000	1.0000	0.25 / 1	0.9816	0.9897	0.9979	0 / 1	2.46	0.007

Table S1 (continued)

		Permanent				LR				DeLong	
Family [b]	Dataset	CI low	AUC	CI high	Sens./spec.[a]	CI low	AUC	CI high	Sens./spec.[a]	Statistic	P
	19	0.9862	0.9945	1.0000	0.15 / 1	0.8960	0.9525	1.0000	0 / 1	1.69	0.046
	20	1.0000	1.0000	1.0000	0.6 / 1	0.9893	0.9946	1.0000	0.05 / 1	1.98	0.024
3	1	0.9814	0.9920	1.0000	0 / 1	0.9243	0.9600	0.9957	0 / 1	2.30	0.011
	2	0.9912	0.9961	1.0000	0 / 1	0.9090	0.9503	0.9916	0 / 1	2.28	0.011
	3	0.9978	0.9992	1.0000	0.15 / 1	0.9648	0.9801	0.9955	0 / 1	2.49	0.006
	4	0.9100	0.9496	0.9892	0 / 1	0.7699	0.8709	0.9719	0 / 1	1.88	0.030
	5	0.9082	0.9449	0.9815	0 / 1	0.8236	0.9062	0.9887	0 / 1	1.13	0.129
	6	0.9895	0.9951	1.0000	0.05 / 1	0.9592	0.9767	0.9942	0 / 1	2.23	0.013
	7	0.9526	0.9738	0.9950	0.05 / 1	0.8185	0.9067	0.9949	0 / 1	1.86	0.031
	8	0.9860	0.9933	1.0000	0 / 1	0.8792	0.9322	0.9853	0 / 1	2.56	0.005
	9	0.9700	0.9874	1.0000	0 / 1	0.8887	0.9443	1.0000	0 / 1	2.14	0.016
	10	0.9870	0.9934	0.9998	0.1 / 1	0.9383	0.9632	0.9880	0.05 / 1	2.83	0.002
	11	0.9624	0.9837	1.0000	0 / 1	0.8771	0.9374	0.9976	0 / 1	2.25	0.012
	12	0.9414	0.9686	0.9958	0.15 / 1	0.9220	0.9521	0.9822	0.05 / 1	1.15	0.126
	13	0.9746	0.9864	0.9983	0 / 1	0.8504	0.9212	0.9919	0 / 1	2.08	0.019
	14	0.9985	0.9995	1.0000	0.05 / 1	0.9669	0.9812	0.9954	0 / 1	2.59	0.005
	15	0.9913	0.9966	1.0000	0 / 1	0.9107	0.9568	1.0000	0 / 1	1.81	0.035
	16	0.9741	0.9858	0.9975	0 / 1	0.8955	0.9447	0.9940	0 / 1	1.93	0.027
	17	0.9878	0.9937	0.9996	0.1 / 1	0.9548	0.9717	0.9887	0 / 1	2.74	0.003
	18	0.8953	0.9458	0.9963	0 / 1	0.9300	0.9559	0.9819	0 / 1	-0.52	0.699
	19	0.9085	0.9472	0.9860	0 / 1	0.8392	0.9059	0.9727	0 / 1	1.38	0.084
	20	1.0000	1.0000	1.0000	0.1 / 1	0.9595	0.9763	0.9931	0 / 1	2.77	0.003
4	1	0.9818	0.9921	1.0000	0 / 1	0.9243	0.9600	0.9957	0 / 1	2.30	0.011
	2	0.9914	0.9962	1.0000	0 / 1	0.9090	0.9503	0.9916	0 / 1	2.27	0.012
	3	0.9975	0.9991	1.0000	0.15 / 1	0.9648	0.9801	0.9955	0 / 1	2.48	0.007
	4	0.9072	0.9482	0.9891	0 / 1	0.7699	0.8709	0.9719	0 / 1	1.87	0.030
	5	0.9088	0.9458	0.9828	0 / 1	0.8236	0.9062	0.9887	0 / 1	1.14	0.127
	6	0.9896	0.9951	1.0000	0.05 / 1	0.9592	0.9767	0.9942	0 / 1	2.23	0.013
	7	0.9525	0.9738	0.9951	0.05 / 1	0.8185	0.9067	0.9949	0 / 1	1.87	0.031
	8	0.9859	0.9932	1.0000	0 / 1	0.8792	0.9322	0.9853	0 / 1	2.55	0.005
	9	0.9714	0.9882	1.0000	0 / 1	0.8887	0.9443	1.0000	0 / 1	2.16	0.016
	10	0.9875	0.9937	0.9999	0.1 / 1	0.9383	0.9632	0.9880	0.05 / 1	2.83	0.002
	11	0.9633	0.9837	1.0000	0 / 1	0.8771	0.9374	0.9976	0 / 1	2.19	0.014
	12	0.9422	0.9689	0.9957	0.15 / 1	0.9220	0.9521	0.9822	0.05 / 1	1.18	0.118
	13	0.9748	0.9864	0.9981	0 / 1	0.8504	0.9212	0.9919	0 / 1	2.08	0.019
	14	0.9987	0.9996	1.0000	0.05 / 1	0.9669	0.9812	0.9954	0 / 1	2.58	0.005
	15	0.9911	0.9964	1.0000	0 / 1	0.9107	0.9568	1.0000	0 / 1	1.81	0.035
	16	0.9735	0.9855	0.9975	0 / 1	0.8955	0.9447	0.9940	0 / 1	1.94	0.026

Table S1 (continued)

Family [b]	Dataset	Permanent				LR				DeLong	
		CI low	AUC	CI high	Sens./spec.[a]	CI low	AUC	CI high	Sens./spec.[a]	Statistic	P
	17	0.9873	0.9934	0.9995	0.1 / 1	0.9548	0.9717	0.9887	0 / 1	2.70	0.003
	18	0.8936	0.9449	0.9962	0 / 1	0.9300	0.9559	0.9819	0 / 1	-0.56	0.712
	19	0.9102	0.9480	0.9858	0 / 1	0.8392	0.9059	0.9727	0 / 1	1.42	0.078
	20	1.0000	1.0000	1.0000	0.05 / 1	0.9595	0.9763	0.9931	0 / 1	2.77	0.003
5	1	0.9425	0.9734	1.0000	0 / 1	0.8667	0.9305	0.9943	0 / 1	2.32	0.010
	2	0.9877	0.9950	1.0000	0 / 1	0.8780	0.9436	1.0000	0 / 1	1.71	0.044
	3	0.9074	0.9554	1.0000	0.05 / 1	0.8427	0.9172	0.9918	0 / 1	2.09	0.018
	4	0.9719	0.9857	0.9994	0 / 1	0.8707	0.9326	0.9946	0 / 1	2.06	0.020
	5	0.9385	0.9657	0.9928	0 / 1	0.8899	0.9367	0.9835	0 / 1	1.55	0.060
	6	0.9725	0.9853	0.9981	0.1 / 1	0.8980	0.9471	0.9962	0.05 / 1	1.79	0.036
	7	0.9023	0.9386	0.9748	0.05 / 1	0.8584	0.9130	0.9677	0 / 1	1.54	0.062
	8	0.9521	0.9787	1.0000	0 / 1	0.8721	0.9317	0.9913	0 / 1	2.36	0.009
	9	0.9759	0.9883	1.0000	0 / 1	0.9392	0.9659	0.9927	0 / 1	1.84	0.033
	10	0.9804	0.9895	0.9986	0.1 / 1	0.9416	0.9655	0.9895	0.1 / 1	2.03	0.021
	11	0.8837	0.9317	0.9797	0 / 1	0.8282	0.9018	0.9755	0 / 1	1.13	0.129
	12	0.9039	0.9593	1.0000	0 / 1	0.8479	0.9263	1.0000	0 / 1	2.13	0.016
	13	0.9772	0.9911	1.0000	0.1 / 1	0.8714	0.9388	1.0000	0 / 1	1.84	0.033
	14	0.9780	0.9879	0.9978	0 / 1	0.8387	0.9154	0.9921	0 / 1	2.00	0.023
	15	0.9684	0.9845	1.0000	0.05 / 1	0.8752	0.9429	1.0000	0 / 1	1.53	0.063
	16	0.9682	0.9817	0.9952	0 / 1	0.9353	0.9596	0.9839	0 / 1	2.07	0.019
	17	0.9786	0.9886	0.9985	0 / 1	0.9446	0.9666	0.9885	0 / 1	2.79	0.003
	18	0.9406	0.9672	0.9939	0 / 1	0.8561	0.9182	0.9802	0 / 1	2.17	0.015
	19	0.9904	0.9954	1.0000	0.1 / 1	0.9410	0.9657	0.9903	0.05 / 1	2.75	0.003
	20	0.8803	0.9337	0.9871	0.05 / 1	0.7289	0.8408	0.9527	0 / 1	2.15	0.016
6	1	0.9411	0.9730	1.0000	0 / 1	0.8667	0.9305	0.9943	0 / 1	2.34	0.010
	2	0.9889	0.9955	1.0000	0.05 / 1	0.8780	0.9436	1.0000	0 / 1	1.70	0.044
	3	0.9039	0.9530	1.0000	0.05 / 1	0.8427	0.9172	0.9918	0 / 1	1.97	0.024
	4	0.9719	0.9857	0.9994	0 / 1	0.8707	0.9326	0.9946	0 / 1	2.07	0.019
	5	0.9374	0.9650	0.9926	0 / 1	0.8899	0.9367	0.9835	0 / 1	1.52	0.065
	6	0.9733	0.9857	0.9980	0.1 / 1	0.8980	0.9471	0.9962	0.05 / 1	1.83	0.034
	7	0.9028	0.9388	0.9749	0.05 / 1	0.8584	0.9130	0.9677	0 / 1	1.53	0.063
	8	0.9526	0.9787	1.0000	0 / 1	0.8721	0.9317	0.9913	0 / 1	2.37	0.009
	9	0.9758	0.9880	1.0000	0 / 1	0.9392	0.9659	0.9927	0 / 1	1.82	0.034
	10	0.9804	0.9895	0.9986	0.1 / 1	0.9416	0.9655	0.9895	0.1 / 1	2.05	0.020
	11	0.8840	0.9318	0.9796	0 / 1	0.8282	0.9018	0.9755	0 / 1	1.15	0.126
	12	0.9025	0.9583	1.0000	0 / 1	0.8479	0.9263	1.0000	0 / 1	2.11	0.018
	13	0.9762	0.9907	1.0000	0.1 / 1	0.8714	0.9388	1.0000	0 / 1	1.84	0.033
	14	0.9789	0.9884	0.9979	0 / 1	0.8387	0.9154	0.9921	0 / 1	2.01	0.022

Table S1 (continued)

Family [b]	Dataset	Permanent				LR				DeLong	
		CI low	AUC	CI high	Sens./spec.[a]	CI low	AUC	CI high	Sens./spec.[a]	Statistic	*P*
	15	0.9672	0.9838	1.0000	0.05 / 1	0.8752	0.9429	1.0000	0 / 1	1.52	0.064
	16	0.9680	0.9817	0.9954	0 / 1	0.9353	0.9596	0.9839	0 / 1	2.08	0.019
	17	0.9784	0.9884	0.9984	0 / 1	0.9446	0.9666	0.9885	0 / 1	2.76	0.003
	18	0.9408	0.9668	0.9929	0 / 1	0.8561	0.9182	0.9802	0 / 1	2.14	0.016
	19	0.9901	0.9953	1.0000	0.1 / 1	0.9410	0.9657	0.9903	0.05 / 1	2.76	0.003
	20	0.8789	0.9330	0.9871	0.05 / 1	0.7289	0.8408	0.9527	0 / 1	2.12	0.017
7	1	0.9985	0.9995	1.0000	0.6 / 1	0.9696	0.9849	1.0000	0.1 / 1	1.95	0.026
	2	1.0000	1.0000	1.0000	0.85 / 1	0.9848	0.9932	1.0000	0.15 / 1	1.60	0.054
	3	1.0000	1.0000	1.0000	0.5 / 1	0.9741	0.9876	1.0000	0 / 1	1.79	0.036
	4	1.0000	1.0000	1.0000	1 / 1	0.9991	0.9997	1.0000	0.1 / 1	0.82	0.205
	5	1.0000	1.0000	1.0000	0.75 / 1	0.9892	0.9950	1.0000	0.15 / 1	1.69	0.045
	6	1.0000	1.0000	1.0000	0.6 / 1	0.9802	0.9908	1.0000	0.05 / 1	1.70	0.045
	7	0.9985	0.9995	1.0000	0.45 / 1	0.9768	0.9870	0.9972	0.1 / 1	2.46	0.007
	8	1.0000	1.0000	1.0000	0.6 / 1	0.9742	0.9866	0.9990	0.15 / 1	2.12	0.017
	9	1.0000	1.0000	1.0000	0.55 / 1	0.9718	0.9861	1.0000	0.2 / 1	1.91	0.028
	10	1.0000	1.0000	1.0000	0.65 / 1	0.9265	0.9714	1.0000	0.15 / 1	1.24	0.107
	11	0.9790	0.9888	0.9986	0.25 / 1	0.9133	0.9546	0.9959	0.05 / 1	1.65	0.050
	12	1.0000	1.0000	1.0000	0.55 / 1	0.9661	0.9859	1.0000	0.15 / 1	1.39	0.082
	13	1.0000	1.0000	1.0000	0.85 / 1	0.9978	0.9992	1.0000	0.05 / 1	1.12	0.131
	14	1.0000	1.0000	1.0000	0.7 / 1	0.9811	0.9908	1.0000	0.1 / 1	1.86	0.031
	15	1.0000	1.0000	1.0000	0.65 / 1	0.9865	0.9934	1.0000	0.15 / 1	1.85	0.032
	16	1.0000	1.0000	1.0000	0.8 / 1	0.9920	0.9963	1.0000	0.15 / 1	1.69	0.045
	17	1.0000	1.0000	1.0000	0.65 / 1	0.9922	0.9964	1.0000	0.2 / 1	1.65	0.049
	18	1.0000	1.0000	1.0000	0.7 / 1	0.9370	0.9770	1.0000	0.05 / 1	1.13	0.129
	19	1.0000	1.0000	1.0000	0.95 / 1	0.9936	0.9972	1.0000	0.25 / 1	1.48	0.070
	20	1.0000	1.0000	1.0000	0.8 / 1	0.9923	0.9964	1.0000	0.05 / 1	1.67	0.047
8	1	0.9964	0.9986	1.0000	0 / 1	0.9500	0.9720	0.9940	0 / 1	2.49	0.006
	2	0.9935	0.9978	1.0000	0.15 / 1	0.8976	0.9579	1.0000	0.05 / 1	1.39	0.082
	3	0.9897	0.9954	1.0000	0.2 / 1	0.8961	0.9461	0.9960	0 / 1	2.14	0.016
	4	0.9985	0.9995	1.0000	0.35 / 1	0.9735	0.9872	1.0000	0.1 / 1	1.81	0.035
	5	0.9973	0.9989	1.0000	0.15 / 1	0.9595	0.9767	0.9939	0.05 / 1	2.65	0.004
	6	0.9414	0.9758	1.0000	0.1 / 1	0.8205	0.9154	1.0000	0 / 1	1.89	0.030
	7	0.9738	0.9861	0.9983	0.2 / 1	0.9355	0.9638	0.9921	0.05 / 1	1.83	0.033
	8	0.9991	0.9997	1.0000	0.2 / 1	0.9410	0.9689	0.9969	0.05 / 1	2.19	0.014
	9	1.0000	1.0000	1.0000	0.2 / 1	0.9598	0.9787	0.9976	0 / 1	2.21	0.014
	10	0.9902	0.9961	1.0000	0.3 / 1	0.9289	0.9675	1.0000	0.05 / 1	1.67	0.048
	11	0.9454	0.9700	0.9946	0.05 / 1	0.8905	0.9358	0.9811	0.05 / 1	1.56	0.060
	12	0.9717	0.9883	1.0000	0.1 / 1	0.8986	0.9482	0.9977	0.05 / 1	2.00	0.023

Table S1 (continued)

Family [b]	Dataset	Permanent				LR				DeLong	
		CI low	AUC	CI high	Sens./spec.[a]	CI low	AUC	CI high	Sens./spec.[a]	Statistic	*P*
	13	1.0000	1.0000	1.0000	0.15 / 1	0.9746	0.9858	0.9969	0 / 1	2.50	0.006
	14	0.9949	0.9979	1.0000	0.05 / 1	0.9176	0.9601	1.0000	0 / 1	1.83	0.034
	15	0.9811	0.9904	0.9997	0.15 / 1	0.8589	0.9334	1.0000	0.05 / 1	1.62	0.053
	16	0.9991	0.9997	1.0000	0.4 / 1	0.9644	0.9817	0.9990	0.05 / 1	2.07	0.019
	17	0.9856	0.9926	0.9996	0.05 / 1	0.8865	0.9486	1.0000	0 / 1	1.50	0.067
	18	0.9991	0.9997	1.0000	0.15 / 1	0.9567	0.9750	0.9933	0 / 1	2.66	0.004
	19	0.9995	0.9999	1.0000	0.35 / 1	0.9665	0.9834	1.0000	0.1 / 1	1.91	0.028
	20	0.9761	0.9866	0.9970	0.05 / 1	0.9229	0.9587	0.9944	0 / 1	1.71	0.043
9	1	0.9748	0.9868	0.9989	0 / 1	0.9214	0.9539	0.9865	0 / 1	2.78	0.003
	2	0.9968	0.9989	1.0000	0.1 / 1	0.9254	0.9612	0.9970	0 / 1	2.16	0.015
	3	0.9743	0.9874	1.0000	0 / 1	0.8701	0.9299	0.9896	0 / 1	2.25	0.012
	4	0.9991	0.9997	1.0000	0.15 / 1	0.9835	0.9913	0.9991	0.05 / 1	2.20	0.014
	5	0.9710	0.9857	1.0000	0.1 / 1	0.9253	0.9553	0.9853	0 / 1	2.71	0.003
	6	0.9224	0.9618	1.0000	0.05 / 1	0.7974	0.8941	0.9907	0 / 1	2.12	0.017
	7	0.9531	0.9753	0.9975	0.05 / 1	0.8923	0.9389	0.9856	0.05 / 1	2.45	0.007
	8	0.9941	0.9978	1.0000	0.1 / 1	0.9469	0.9683	0.9897	0.05 / 1	2.91	0.002
	9	0.9948	0.9978	1.0000	0.05 / 1	0.9319	0.9607	0.9895	0 / 1	2.61	0.005
	10	0.9931	0.9971	1.0000	0.1 / 1	0.9111	0.9601	1.0000	0.05 / 1	1.58	0.057
	11	0.9194	0.9516	0.9837	0.05 / 1	0.8310	0.8962	0.9614	0.05 / 1	1.85	0.032
	12	0.9627	0.9846	1.0000	0.1 / 1	0.8948	0.9432	0.9915	0.05 / 1	2.37	0.009
	13	1.0000	1.0000	1.0000	0.05 / 1	0.9640	0.9796	0.9952	0 / 1	2.56	0.005
	14	0.9857	0.9928	0.9999	0 / 1	0.8796	0.9413	1.0000	0 / 1	1.78	0.037
	15	0.9554	0.9759	0.9964	0.1 / 1	0.8431	0.9182	0.9932	0 / 1	1.80	0.036
	16	0.9985	0.9995	1.0000	0.25 / 1	0.9674	0.9836	0.9997	0 / 1	2.01	0.022
	17	0.9716	0.9854	0.9992	0.05 / 1	0.9027	0.9446	0.9865	0 / 1	2.15	0.016
	18	0.9995	0.9999	1.0000	0.1 / 1	0.9315	0.9593	0.9872	0 / 1	2.86	0.002
	19	0.9949	0.9978	1.0000	0.15 / 1	0.9546	0.9764	0.9983	0 / 1	2.04	0.020
	20	0.9953	0.9980	1.0000	0 / 1	0.9162	0.9580	0.9998	0 / 1	1.89	0.029
10	1	0.7801	0.8582	0.9362	0 / 1	0.7735	0.8455	0.9175	0 / 1	0.52	0.300
	2	0.8294	0.8975	0.9656	0 / 1	0.7655	0.8492	0.9329	0 / 1	2.00	0.023
	3	0.6426	0.7587	0.8748	0 / 1	0.5843	0.7168	0.8494	0 / 1	1.56	0.060
	4	0.7018	0.8001	0.8985	0 / 1	0.6393	0.7518	0.8644	0 / 1	1.90	0.029
	5	0.7973	0.8666	0.9358	0 / 1	0.7090	0.8145	0.9199	0 / 1	2.08	0.019
	6	0.8687	0.9245	0.9803	0 / 1	0.7820	0.8713	0.9606	0 / 1	2.10	0.018
	7	0.5543	0.7014	0.8486	0 / 1	0.5718	0.6988	0.8258	0 / 1	0.05	0.478
	8	0.7445	0.8332	0.9218	0 / 1	0.7526	0.8254	0.8982	0 / 1	0.28	0.389
	9	0.6529	0.7632	0.8734	0 / 1	0.6405	0.7518	0.8632	0 / 1	0.53	0.297
	10	0.7214	0.8067	0.8920	0 / 1	0.6885	0.7839	0.8794	0 / 1	0.74	0.229

Table S1 (continued)

Family [b]	Dataset	Permanent				LR				DeLong	
		CI low	AUC	CI high	Sens./spec.[a]	CI low	AUC	CI high	Sens./spec.[a]	Statistic	P
	11	0.5973	0.7404	0.8835	0 / 1	0.6049	0.7430	0.8812	0 / 1	-0.08	0.531
	12	0.6726	0.7854	0.8982	0 / 1	0.6120	0.7397	0.8675	0 / 1	2.95	0.002
	13	0.8291	0.9116	0.9941	0 / 1	0.8233	0.8889	0.9546	0 / 1	1.30	0.096
	14	0.5191	0.6537	0.7882	0 / 1	0.4807	0.6295	0.7783	0 / 1	0.73	0.232
	15	0.7695	0.8479	0.9263	0 / 1	0.7547	0.8254	0.8960	0 / 1	1.03	0.152
	16	0.7213	0.8225	0.9237	0 / 1	0.6567	0.7751	0.8935	0 / 1	1.56	0.059
	17	0.6575	0.7507	0.8438	0 / 1	0.6225	0.7275	0.8325	0 / 1	0.73	0.234
	18	0.8171	0.8862	0.9552	0 / 1	0.7686	0.8520	0.9354	0 / 1	1.10	0.135
	19	0.8600	0.9149	0.9698	0 / 1	0.7930	0.8728	0.9526	0 / 1	2.04	0.021
	20	0.7013	0.8305	0.9597	0 / 1	0.6661	0.7974	0.9287	0 / 1	2.65	0.004

[a]Sensitivity and specificity at threshold 0.999

[b]Family types tested. Definitions of the family types are shown in Table 1

Permanent: permanent method; LR: LR method; AUC: area under curve; CI low: 95% confidence interval of AUC, lower bound; CI high: 95% confidence interval of AUC, upper bound

Table S2: Counts of family types in each mixed dataset

Dataset	Family type[a]										Total
	1	2	3	4	5	6	7	8	9	10	
1	1	2	2	2	3	0	2	5	1	2	20
2	5	2	3	2	2	0	2	2	0	2	20
3	1	3	2	2	1	2	1	4	1	3	20
4	1	0	1	3	4	4	3	2	0	2	20
5	2	5	2	1	2	2	2	0	1	3	20
6	2	1	2	6	3	2	1	0	0	3	20
7	1	0	4	6	2	1	1	0	2	3	20
8	2	2	1	2	3	2	2	3	2	1	20
9	3	1	2	2	2	2	2	1	0	5	20
10	0	2	3	1	1	5	1	3	2	2	20
11	3	2	2	0	0	2	4	3	2	2	20
12	2	1	0	3	4	1	2	3	1	3	20
13	1	1	2	2	2	2	5	2	2	1	20
14	1	1	2	2	3	3	1	1	4	2	20
15	1	5	4	1	1	3	1	2	0	2	20
16	2	1	1	2	1	2	0	3	6	2	20
17	2	2	3	4	5	1	2	1	0	0	20
18	1	1	4	1	2	1	1	5	3	1	20
19	1	4	1	4	4	0	2	1	2	1	20
20	2	1	5	4	0	2	1	4	0	1	20
Total	34	37	46	50	45	37	36	45	29	41	400

[a]A list of typed and missing individuals for each family type is shown in Table 1

Table S3: Result of each mixed dataset

	Permanent				LR				DeLong	
Dataset	CI low	AUC	CI high	Sens./spec.[a]	CI low	AUC	CI high	Sens./spec.[a]	Statistic	P
1	1.0000	1.0000	1.0000	0.1 / 1	0.9429	0.9713	0.9997	0 / 1	1.98	0.024
2	0.9983	0.9995	1.0000	0.4 / 1	0.9756	0.9878	1.0000	0.1 / 1	1.93	0.027
3	1.0000	1.0000	1.0000	0.15 / 1	0.9187	0.9654	1.0000	0 / 1	1.45	0.073
4	0.9538	0.9759	0.9980	0.1 / 1	0.9286	0.9567	0.9848	0.05 / 1	1.90	0.029
5	0.9947	0.9980	1.0000	0.1 / 1	0.9351	0.9636	0.9921	0 / 1	2.59	0.005
6	0.9564	0.9775	0.9986	0.2 / 1	0.8807	0.9399	0.9990	0 / 1	1.24	0.107
7	0.9432	0.9668	0.9905	0.05 / 1	0.8353	0.9043	0.9734	0 / 1	2.42	0.008
8	0.9729	0.9854	0.9979	0.1 / 1	0.8855	0.9358	0.9861	0 / 1	2.16	0.015
9	0.9338	0.9626	0.9915	0.25 / 1	0.9137	0.9486	0.9834	0.2 / 1	1.22	0.111
10	0.9448	0.9725	1.0000	0.15 / 1	0.8749	0.9322	0.9895	0.1 / 1	2.17	0.015
11	0.9333	0.9686	1.0000	0.2 / 1	0.9191	0.9579	0.9967	0.15 / 1	0.65	0.259
12	0.9738	0.9880	1.0000	0.2 / 1	0.9156	0.9574	0.9992	0.05 / 1	2.02	0.022
13	0.9952	0.9984	1.0000	0.15 / 1	0.9212	0.9692	1.0000	0 / 1	1.27	0.101
14	0.9539	0.9771	1.0000	0.05 / 1	0.8493	0.9189	0.9886	0.05 / 1	2.34	0.010
15	0.9934	0.9975	1.0000	0.15 / 1	0.8992	0.9591	1.0000	0.05 / 1	1.34	0.090
16	1.0000	1.0000	1.0000	0.25 / 1	0.9661	0.9838	1.0000	0.15 / 1	1.79	0.037
17	0.9857	0.9925	0.9993	0.35 / 1	0.9701	0.9824	0.9946	0.1 / 1	1.93	0.027
18	0.9881	0.9939	0.9998	0.25 / 1	0.9601	0.9757	0.9912	0.05 / 1	2.68	0.004
19	0.9976	0.9991	1.0000	0.1 / 1	0.9489	0.9711	0.9932	0 / 1	2.54	0.006
20	1.0000	1.0000	1.0000	0.45 / 1	0.9508	0.9779	1.0000	0.05 / 1	1.60	0.055

[a]Sensitivity and specificity at threshold 0.999

Permanent: permanent method; LR: LR method; AUC: area under curve; CI low: 95% confidence interval of AUC, lower bound; CI high: 95% confidence interval of AUC, upper bound

[a]Sensitivities and specificities at $v = 0.999$

Table S4: Counts of family types in the complete part and each additional part

Situation	Number of added data	Dataset	Family type 1	2	3	4	5	6	7	8	9	10	Total
Complete			3	2	1	4	1	2	4	1	0	2	20
1	4	1	1	0	0	1	0	0	1	0	1	0	4
		2	1	0	0	1	0	0	0	1	1	0	4
		3	1	0	0	0	0	1	0	2	0	0	4
		4	1	0	0	1	0	0	1	0	0	1	4
		5	1	0	0	0	0	0	1	0	0	2	4
		6	0	0	0	1	1	0	0	1	1	0	4
		7	1	0	2	0	0	0	0	1	0	0	4
		8	0	1	0	0	0	2	0	0	1	0	4
		9	0	0	0	1	1	0	1	0	0	1	4
		10	1	0	0	0	0	0	2	1	0	0	4
	10	1	2	2	3	0	1	0	1	0	0	1	10
		2	1	1	0	1	1	3	0	2	0	1	10
		3	0	0	2	1	3	1	0	1	1	1	10
		4	2	1	0	0	0	5	0	0	1	1	10
		5	3	0	0	2	2	0	0	3	0	0	10
		6	2	0	0	0	0	3	1	2	1	1	10
		7	2	2	2	1	1	0	1	0	1	0	10
		8	0	1	1	0	2	1	2	0	2	1	10
		9	1	1	1	0	2	2	2	1	0	0	10
		10	0	0	0	2	2	1	3	0	1	1	10
2	4	1	0	0	0	0	1	1	0	1	1	0	4
		2	0	0	3	0	0	0	0	1	0	0	4
		3	0	0	1	0	0	0	1	0	1	1	4
		4	0	0	0	1	1	1	0	0	0	1	4
		5	0	0	0	3	0	0	0	0	0	1	4
		6	0	1	1	1	0	0	0	0	1	0	4
		7	1	0	0	0	1	1	0	0	0	1	4
		8	0	1	1	1	0	0	0	0	1	0	4
		9	0	1	0	0	1	0	0	0	1	1	4
		10	1	0	0	1	0	0	0	0	0	2	4
	10	1	1	0	0	0	1	0	1	0	4	3	10
		2	1	1	1	2	0	2	1	2	0	0	10
		3	0	0	2	2	1	3	0	1	0	1	10
		4	1	2	2	2	0	1	0	2	0	0	10
		5	1	4	1	2	0	0	1	0	0	1	10
		6	2	0	2	0	0	1	2	0	1	2	10
		7	0	1	2	3	1	0	1	0	1	1	10
		8	2	1	1	2	1	0	0	1	2	0	10
		9	1	1	3	0	0	0	2	1	1	1	10
		10	1	2	1	1	0	1	0	2	1	1	10
3	4	1	0	1	0	0	1	0	0	1	1	0	4
		2	0	0	1	1	1	0	0	0	0	1	4
		3	0	0	1	0	1	0	2	0	0	0	4
		4	0	1	1	0	1	0	0	1	0	0	4

Table S4 (continued)

Situation	Number of added data	Dataset	Family type 1	2	3	4	5	6	7	8	9	10	Total
		5	0	0	1	0	1	0	1	1	0	0	4
		6	1	0	0	0	0	1	1	0	1	0	4
		7	0	0	0	1	3	0	0	0	0	0	4
		8	0	0	1	0	0	0	0	3	0	0	4
		9	0	0	0	0	2	0	0	0	1	1	4
		10	0	0	2	0	0	0	1	0	0	1	4
	10	1	1	1	3	0	0	1	0	3	1	0	10
		2	0	1	3	0	1	1	1	1	1	1	10
		3	2	0	1	1	0	2	1	1	0	2	10
		4	1	1	2	1	0	1	1	0	2	1	10
		5	1	0	0	2	2	2	0	0	1	2	10
		6	1	1	0	0	1	2	3	0	2	0	10
		7	0	0	1	2	0	2	2	1	2	0	10
		8	0	2	1	1	0	1	0	2	2	1	10
		9	0	1	1	1	1	1	0	3	1	1	10
		10	2	1	2	1	0	1	1	1	0	1	10
		Total	44	36	54	48	40	47	43	45	41	42	440

Table S5: Results of each dataset with unavailable data

Situation	No. of additional data	Dataset	CI low	AUC	CI high	Decrease[a]	Sens./spec.[b]
Complete			0.9887	0.9945	1.0000		0.25/1
1	4	1	0.9852	0.9928	1.0000	0.0017	0.05 / 1
		2	0.9866	0.9934	1.0000	0.0011	0.05 / 1
		3	0.9882	0.9943	1.0000	0.0001	0.05 / 1
		4	0.9875	0.9939	1.0000	0.0005	0.05 / 1
		5	0.9871	0.9939	1.0000	0.0005	0.05 / 1
		6	0.9856	0.9933	1.0000	0.0012	0.05 / 1
		7	0.9848	0.9936	1.0000	0.0009	0.15 / 1
		8	0.9862	0.9936	1.0000	0.0009	0.05 / 1
		9	0.9874	0.9941	1.0000	0.0004	0.05 / 1
		10	0.9873	0.9939	1.0000	0.0005	0.05 / 1
	10	1	0.9816	0.9917	1.0000	0.0028	0.05 / 1
		2	0.9748	0.9899	1.0000	0.0046	0.05 / 1
		3	0.9812	0.9918	1.0000	0.0026	0.05 / 1
		4	0.9816	0.9916	1.0000	0.0029	0.05 / 1
		5	0.9863	0.9934	1.0000	0.0011	0.05 / 1
		6	0.9761	0.9904	1.0000	0.0041	0.05 / 1
		7	0.9835	0.9918	1.0000	0.0026	0.05 / 1
		8	0.9789	0.9895	1.0000	0.0050	0.05 / 1
		9	0.9761	0.9892	1.0000	0.0053	0.05 / 1
		10	0.9856	0.9933	1.0000	0.0012	0.05 / 1
2	4	1	0.9731	0.9880	1.0000	0.0064	0.2 / 1
		2	0.9867	0.9937	1.0000	0.0008	0.15 / 1
		3	0.9862	0.9932	1.0000	0.0013	0.1 / 1

Table S5 (continued)

Situation	No. of additional data	Dataset	CI low	AUC	CI high	Decrease[a]	Sens./spec.[b]
		4	0.9821	0.9914	1.0000	0.0030	0.1 / 1
		5	0.9873	0.9939	1.0000	0.0005	0.1 / 1
		6	0.9854	0.9929	1.0000	0.0016	0.15 / 1
		7	0.9859	0.9932	1.0000	0.0013	0.2 / 1
		8	0.9860	0.9934	1.0000	0.0011	0.15 / 1
		9	0.9875	0.9938	1.0000	0.0007	0.1 / 1
		10	0.9879	0.9942	1.0000	0.0003	0.1 / 1
	10	1	0.9813	0.9914	1.0000	0.0030	0.05 / 1
		2	0.9782	0.9901	1.0000	0.0043	0.1 / 1
		3	0.9809	0.9914	1.0000	0.0030	0.1 / 1
		4	0.9798	0.9911	1.0000	0.0034	0.1 / 1
		5	0.9804	0.9913	1.0000	0.0032	0.1 / 1
		6	0.9703	0.9863	1.0000	0.0082	0.1 / 1
		7	0.9835	0.9921	1.0000	0.0024	0.05 / 1
		8	0.9834	0.9924	1.0000	0.0021	0.1 / 1
		9	0.9816	0.9913	1.0000	0.0032	0.05 / 1
		10	0.9810	0.9909	1.0000	0.0036	0.1 / 1
3	4	1	0.9829	0.9925	1.0000	0.0020	0.05 / 1
		2	0.9828	0.9922	1.0000	0.0022	0.05 / 1
		3	0.9862	0.9936	1.0000	0.0009	0.05 / 1
		4	0.9854	0.9933	1.0000	0.0012	0.05 / 1
		5	0.9763	0.9893	1.0000	0.0051	0.05 / 1
		6	0.9774	0.9901	1.0000	0.0043	0.05 / 1
		7	0.9857	0.9932	1.0000	0.0013	0.05 / 1
		8	0.9848	0.9933	1.0000	0.0012	0.05 / 1
		9	0.9796	0.9896	0.9996	0.0049	0.05 / 1
		10	0.9844	0.9926	1.0000	0.0018	0.05 / 1
	10	1	0.9794	0.9908	1.0000	0.0037	0.05 / 1
		2	0.9765	0.9887	1.0000	0.0058	0.05 / 1
		3	0.9680	0.9864	1.0000	0.0080	0.05 / 1
		4	0.9718	0.9871	1.0000	0.0074	0.05 / 1
		5	0.9816	0.9914	1.0000	0.0030	0.05 / 1
		6	0.9817	0.9912	1.0000	0.0033	0.05 / 1
		7	0.9792	0.9903	1.0000	0.0042	0.05 / 1
		8	0.9729	0.9883	1.0000	0.0062	0.05 / 1
		9	0.9385	0.9757	1.0000	0.0188	0.05 / 1
		10	0.9825	0.9918	1.0000	0.0026	0.05 / 1

[a]Decrease of the AUC of the incomplete data from that of the complete data
[b]Sensitivities and specificities at threshold 0.999
AUC: area under curve; CI low: 95% confidence interval of AUC, lower bound; CI high: 95% confidence interval of AUC, upper bound;

Table S6: Acceptance number, k

	ϵ				
δ	0.1	0.01	0.001	0.0001	0.00001
1	42	75	107	139	171
0.5	168	297	426	555	684
